# The covalent modification of STAT1 cysteines by sulforaphane promotes antitumor immunity via blocking IFN-γ-induced PD-L1 expression

**DOI:** 10.1016/j.redox.2025.103543

**Published:** 2025-02-11

**Authors:** Qing Shi, Yajuan Liu, Wanqi Yang, Yao Li, Chenji Wang, Kun Gao

**Affiliations:** a State Key Laboratory of Genetic Engineering, Shanghai Stomatological Hospital & School of Stomatology, MOE Engineering Research Center of Gene Technology, Shanghai Engineering Research Center of Industrial Microorganisms, School of Life Sciences, Fudan University, Shanghai, 200438, China; b Department of Clinical Laboratory, Shanghai First Maternity and Infant Hospital, School of Medicine, Tongji University, Shanghai, 200092, China; c Shanghai Key Laboratory of Maternal and Fetal Medicine, Shanghai First Maternity and Infant Hospital, Shanghai, 200092, China; d Shanghai Key Laboratory of Metabolic Remodeling and Health, Institute of Metabolism and Integrative Biology, Centre for Evolutionary Biology, Fudan, Fudan University, Shanghai, 200438, China

**Keywords:** Sulforaphane, STAT1, PD-L1, Antitumor immunity

## Abstract

Sulforaphane (SFN), a natural compound found in cruciferous vegetables, possesses well-documented antitumor properties. However, the precise functions and mechanisms of SFN in cancer suppression remain poorly understood. Here we provide evidence to demonstrate that SFN exerts more pronounced antitumor effects in immunocompetent mice compared to immunodeficient mice, suggesting the involvement of the host immune system in SFN-mediated tumor suppression. Furthermore, we reveal that SFN primarily acts through CD8^+^ cytotoxic T lymphocytes (CTLs) to enhance antitumor immunity by blocking the IFN-γ-mediated induction of PD-L1, a critical immune checkpoint receptor expressed in cancer cells. Importantly, our findings indicate that the suppression of PD-L1 expression by SFN is independent of the NRF2 protein stabilization pathway. Instead, SFN inhibits IFN-γ-mediated activation of STAT1, a key transcription factor involved in PD-L1 induction. Mechanistically, SFN covalently modifies specific cysteine residues (C155 and C174) on STAT1, resulting in the inhibition of its transcriptional activity. Notably, SFN-mediated downregulation of PD-L1 contributes to its antitumor immune effects, as demonstrated by enhanced anti-CTLA-4-mediated cytotoxicity. These findings indicate that SFN's antitumor effect extends beyond its direct cytotoxic properties, as it also actively engages the host immune system. This underscores SFN's immense potential as an immune-modulating agent in cancer therapy.

## Highlights

• Sulforaphane enhances CD8^+^ T cell-mediated cytotoxicity, boosting antitumor immunity.
• Sulforaphane acts independently of the Keap1-NRF2 pathway to suppress PD-L1 expression.
• Sulforaphane inhibits PD-L1 expression via covalent modification of STAT1 cysteine residues.
• Combining sulforaphane with anti-CTLA-4 therapy improves tumor suppression *in vivo.*

## Graphical abstract

**Figure:**
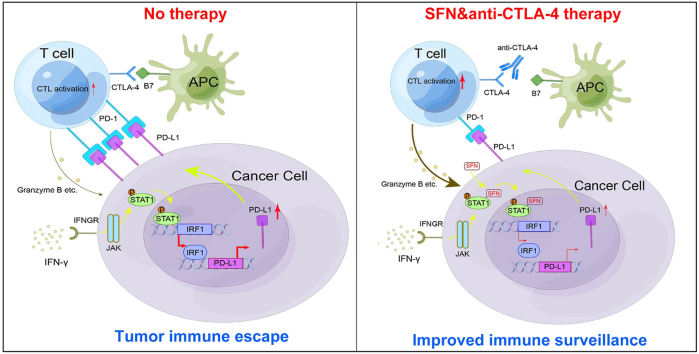

## 1 Introduction

Epidemiological studies have consistently shown that consuming cruciferous vegetables, including broccoli, kale, cabbage, Brussels sprouts, garden cress, watercress, and cauliflower, is associated with a significant reduction in the risk of various cancers [1,2]. These vegetables, belonging to the Brassicaceae plant family, are not only rich in nutrients such as carotenoids, flavonoids, anthocyanins, coumarins, terpenes, vitamins, folate, and fiber but also contain sulfur-containing glucosinolates. When broken down by myrosinase enzymes or β-thioglucosidases in gut bacteria, these glucosinolates produce biologically active compounds known as isothiocyanates (ITCs) [3]. Among the ITCs, sulforaphane (SFN) has garnered considerable attention in cancer research due to its potent chemopreventive properties demonstrated in cell cultures, animal models, and clinical trials [4]. SFN is capable of inducing a range of cellular processes that can effectively impede the initiation, promotion, and progression of cancer, making it a focal point in oncology [5].

Previous studies have significantly advanced our understanding of the molecular mechanisms underlying the bioactivity of SFN [5]. These investigations have primarily focused on elucidating the selective targeting of specific substrates, with particular emphasis on Keap1, which has emerged as one of the most extensively studied molecules in this context. Keap1 serves as a key focal point due to its crucial role as an adaptor of the Cullin 3-RING E3 ligase (CRL3) complex [6]. Through this role, Keap1 facilitates the ubiquitin-proteasomal degradation of the transcription factor NRF2 [7]. The comprehensive examination of Keap1 has illuminated its intricate interactions with SFN, an electrophilic agent capable of covalently modifying cysteine residues, specifically at position 151 on Keap1[8]. This covalent modification induces a conformational change in Keap1, which disrupts its inhibitory function and subsequently triggers the activation of NRF2. Consequently, this intricate molecular cascade initiates the expression of cytoprotective genes involved in antioxidative and anti-inflammatory responses, as well as the upregulation of detoxifying enzymes [9]. These findings underscore the importance of Keap1 as a central player in SFN's bioactivity.

The co-inhibitory PD-1 pathway has garnered significant attention for its critical role modulating the immune checkpoint response of CTLs, allowing tumor cells to evade immune surveillance [10]. The development of therapeutic antibodies targeting the PD-1 pathway, specifically PD1 or its ligand PD-L1, has led to groundbreaking advancements in cancer therapeutics. While checkpoint blockade immunotherapy has demonstrated notable efficacy in certain cancer types, the response rates among patients exhibit substantial variation, with only a small subset experiencing a favorable response within a large cohort [11]. Generally, PD-L1 expression correlates with clinical response to PD-1/PD-L1 therapy [12,13]. PD-L1 expression is regulated at multiple levels, encompassing transcriptional, translational, and post-translational mechanisms. The induction of PD-L1 expression is primarily regulated by interferon regulatory factor-1 (IRF1), a key transcription factor that operates through the IFN-γ-driven JAK/STAT-IRF1 signaling cascade [14].

In this study, we unexpectedly observed the inhibitory effects of SFN on IFN-γ-inducible PD-L1 expression in cancer cells. Furthermore, we conducted meticulous examinations of SFN's antitumor immune response both *in vivo* and *in vitro* using multiple cancer cell models. Additionally, we evaluated the potential of SFN as an adjunct to immunotherapy to enhance the efficacy of current therapeutic strategies.

## 2 Results

### 2.1 SFN exert an antitumor activity via the host immune system

**Fig. 1:**
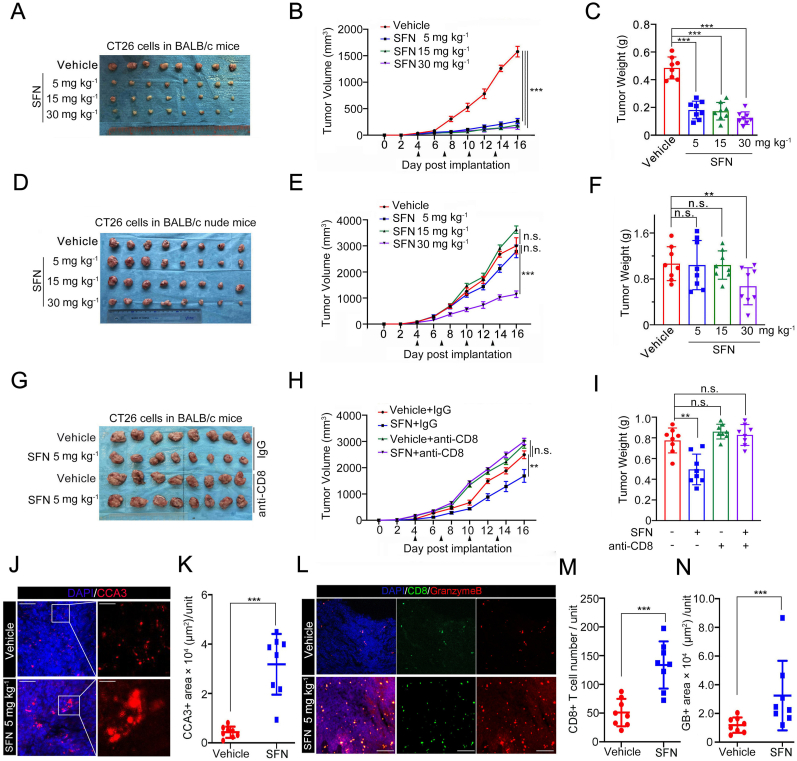
SFN exert an antitumor activity through the host immune system. **A-C** CT26 cells were injected s.c. into the right flank of BALB/c mice and treated with vehicle or SFN at different day points. Tumor growth was measured every other day for 16 days. Tumors in each group at day 16 were harvested and photographed (A), tumor volume (B) and tumor weight (C) at each time point was documented. n = 8 (8 mice per group). **D-F** CT26 cells were injected s.c. into the right flank of immunodeficient nude mice and treated with vehicle or SFN at different day points. Tumor growth was measured every other day for 16 days. Tumors in each group at day 16 were harvested and photographed (D), tumor volume (E) and tumor weight (F) at each time point was documented. n = 8 (8 mice per group). **G-I** CT26 cells were injected s.c. into the right flank of BALB/c mice and treated with SFN or IgG, anti-CD8 antibody at different day points. Tumor growth was measured every other day for 16 days. Tumors in each group at day 16 were harvested and photographed (G), tumor volume (H) and tumor weight (I) at each time point was documented. n = 8 (8 mice per group). **J** Immunostaining of cleaved caspase 3(CCA3) in the CT26 tumors shown in (Fig. 1, Fig. 5 mg kg-1, group). DAPI: nuclear. Counterstaining. Scale bar, 150 μm. (80 μm in inset). **K** Quantification of CCA3 using imageJ. n = 8 (8 mice per group). Unit = 372943 μm^2^ **l** Immunostaining of CD8 and Granzyme B in the CT26 tumors shown in (Fig. 1A, DMSO and 5 mg kg^−1^, group). DAPI: nuclear. Counterstaining. Scale bar, 200 μm. **M,N** Quantification of CD8 (M) and Granzyme B(N) using imageJ. n = 8 (8 mice per group). Unit = 660304 μm^2^. The *p* values were calculated using the Two-way ANOVA test in (B, E, H). The *p* values were calculated using the One-way ANOVA test in (C, F, I, K, M, N). *∗p* < 0.05, *∗∗p* < 0.01, *∗∗∗p* < 0.001.

We conducted a comparative analysis to assess the impact of SFN on tumor growth in the presence or absence of a functional immune system. To investigate this, we employed murine CT26 colon carcinoma cells, which are commonly utilized in immunocompetent mouse models to study tumor immune escape. Three different doses of SFN (low, intermediate, and high) were administered, and even at a low dose of 5 mg kg^−1^, SFN demonstrated a significant antitumor effect on CT26 tumors grown in immunocompetent C57BL/6 mice (Fig. 1A–C). In contrast, when tested in immunodeficient nude mice, only the high dose of SFN treatment exhibited an obvious antitumor effect, highlighting the critical role of the host immune system in SFN's antitumor response (Fig. 1D–F). Furthermore, we performed similar assays in C57BL/6 mice depleted of CD8^+^ CTLs using an anti-CD8 antibody, revealing that the antitumor activity of SFN is predominantly mediated by CD8^+^ CTLs, the primary effectors of antitumor immunity (Fig. 1G–I, Figs. S1A–C). Since antitumor immunity is often accompanied by apoptosis in tumor tissues, we compared levels of cleaved caspase 3 (CCA3) and observed stronger apoptotic signals in the tumors from SFN-treated group compared to the control group (Fig. 1J and K). Given that CD8^+^ CTLs eliminate cancer cells by secreting granzyme B (GB), a potent inducer of tumor cell apoptosis, we investigated the CD8^+^ CTL population and CTL activity by measuring GB release. Indeed, SFN treatment significantly increased both the CD8^+^ CTL population and GB release compared to the control group (Fig. 1L–N). Collectively, our results indicate that SFN exerts its antitumor effects by enhancing CTL activity in addition to its direct antitumor activity on cancer cells.

### 2.2 SFN blocks IFN-γ-inducible PD-L1 expression in human and mouse cancer cells

**Fig. 2:**
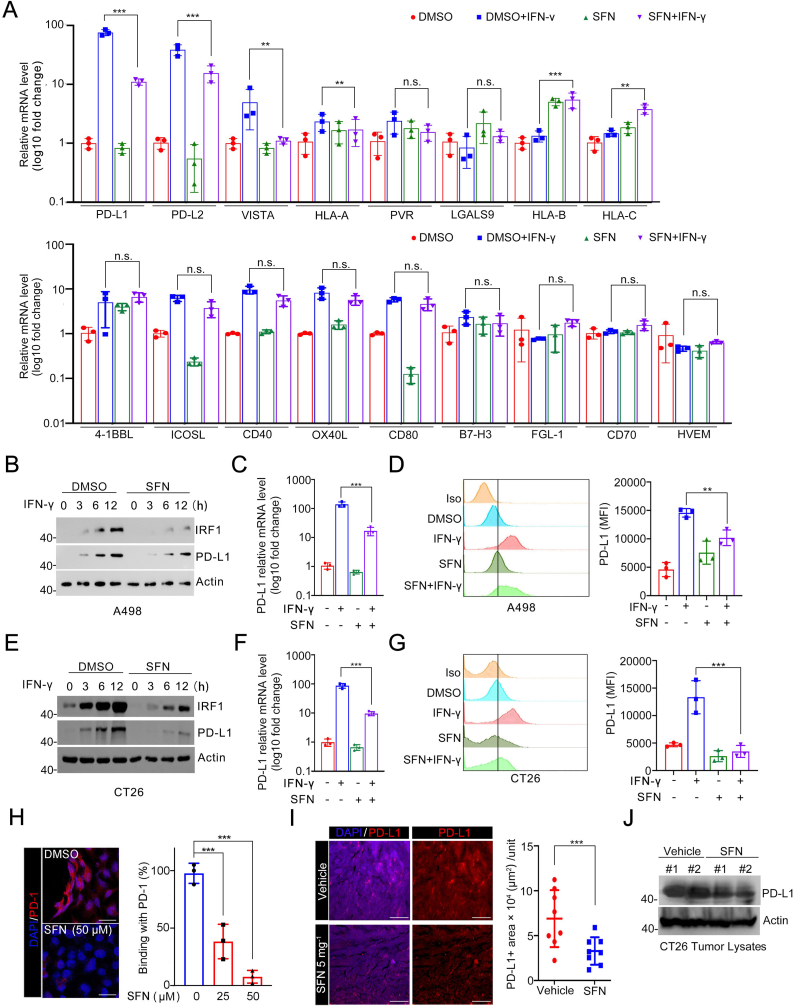
SFN blocks IFN-γ-inducible PD-L1 expression. **A** RT-qPCR analyses of immune checkpoint receptors and HLA-I mRNA levels in A498 cells were pretreated with SFN (25 μM) for 2 h and then treated with DMSO or IFN-γ (200 ng/mL) for 10 h. The mRNA level of GAPDH was used for normalization. n = 3. **B** WB analyses of the indicated proteins in A498 cells pretreated with SFN (25 μM) for 2 h and then treated with DMSO or IFN-γ (200 ng/mL) for the indicated times. **C** RT-qPCR analyses of PD-L1 mRNA levels in A498 cells pretreated with SFN (25 μM) for 2 h and then treated with DMSO or IFN-γ (200 ng/mL) for 10 h n = 3. **D** Flow cytometry analyses of surface PD-L1 in A498 cells pretreated with SFN (25 μM) for 2 h and then treated with DMSO or IFN-γ (200 ng/mL) for 10 h n = 3. MFI: Mean Fluorescent Intensity. **E** WB analyses of the indicated proteins in CT26 cells pretreated with SFN (25 μM) for 2 h and treated with DMSO or IFN-γ (200 ng/mL) for the indicated times. **F** RT-qPCR analyses of PD-L1 mRNA levels in CT26 cells pretreated with SFN (25 μM) for 2 h and treated with DMSO or IFN-γ (200 ng/mL) for 10 h n = 3. **G** Flow cytometry analyses of surface PD-L1 in CT26 cells pretreated with SFN (25 μM) for 2 h and then treated with DMSO or IFN-γ (200 ng/mL) for 10 h n = 3. **H** Quantitation of binding of PD-1 to PD-L1 in A498 cells treated with DMSO or SFN (50 μM) for 12 h (left panel). n = 3. Representative images are shown (right panel). Scale bar, 25 μm. **I** Immunostaining of PD-L1 in the CT26 tumors (Fig. 1A, DMSO and 5 mg kg^−1^ SFN group, left panel). DAPI: nuclear. Counterstaining. Scale bar, 200 μm. Quantification of PD-L1 using imageJ (right panel). n = 8 (8 mice per group). Unit = 660304 μm^2^. **J** WB analyses of the indicated proteins in the WCL extracted from the tumor tissues (Fig. 1A, DMSO and 5 mg kg^−1^, group).

To unravel the molecular mechanisms underlying SFN's antitumor immune effect, we investigated its impact on the expression of immune checkpoint receptors and class I human leukocyte antigens (HLA-I), known to play an critical role in tumor immune evasion [15,16]. Considering that IFN-γ can transcriptionally induce the expression of multiple immune checkpoint receptors, we examined the mRNA levels of immune checkpoint regulators under basal conditions and IFN-γ-treated conditions. Generally, SFN did not show a significant effect on the mRNA levels of the immune checkpoint receptors examined under basal conditions in A498 cells. However, we observed a significant suppression of IFN-γ-induced upregulation of PD-L1, PD-L2, HLA-A, and VISTA by SFN treatment, while other immune checkpoint regulators are minimally affected (Fig. 2A). Given the remarkable success of PD-1/PD-L1 blockade in cancer treatment, we investigated whether SFN exerts its antitumor immune effect by inhibiting IFN-γ-induced PD-L1 upregulation.

Given the broad-spectrum anticancer and preventive properties of SFN, we evaluated the inhibitory effects of SFN on IFN-γ-induced PD-L1 expression in a diverse range of tumor types, rather than focusing solely on a single tumor type. To assess PD-L1 expression, we employed Western blotting (WB) for protein level, quantitative reverse transcription-polymerase chain reaction (qRT-PCR) for mRNA level, and flow cytometry for cell surface level. Consistently, these analyses demonstrated that SFN effectively blocked PD-L1 induction in multiple human cancer cell lines, including A498 kidney cancer cells (Fig. 2B–D), H1299 lung cancer cells (Figs. S2A–C), and DU145 prostate cancer cells (Figs. S2D–F). Similarly, SFN efficiently blocked PD-L1 induction in multiple murine cancer cell lines, including CT26 colon cancer cells (Fig. 2E–G), LLC lung cancer cells (Figs. S2G–I), and KPIC pancreatic cancer cells (Figs. S2J–L). PD-1/PD-L1 binding assay analysis revealed significantly reduced cell membrane PD-L1 levels, leading to decreased binding with PD-1 (Fig. 2H). Immunofluorescence (IF) and WB analyses further confirmed a marked decrease in PD-L1 expression in CT26 tumors following SFN treatment (Fig. 2I and J). Prior to initiating this treatment, we conducted a series of tests using escalating concentrations of SFN and determined the optimal concentration (25 μM) that effectively blocked IFN-γ-inducible PD-L1 while avoiding apoptosis (Figs. S3A and B). Furthermore, we investigated the role of IRF1, a primary transcription factor responsible for PD-L1 induction through the IFN-γ-driven JAK/STAT-IRF1 signaling cascade[14]. SFN not only blocked IFN-γ-induced PD-L1 expression but also IFN-γ-induced IRF1 expression, indicating that SFN acts upstream of IRF1 to inhibit PD-L1 induction (Fig. 2B–G, Figs. S2A–I).

Finally, we examined several naturally occurring ITCs analogous to SFN, including Erucin, Iberin, Cheriolin, and Berteroin (Fig. S4A)[17]. Our results showed that, with the exception of Erucin, other ITCs also suppressed IFN-γ-induced expression of PD-L1 and IRF1, albeit to varying degrees (Figs. S4B–D). Collectively, our results indicate that SFN effectively blocks IFN-γ-inducible PD-L1 and IRF1 expression in various human and murine cellular models.

### 2.3 SFN blocks PD-L1 expression independent of its roles towards the Keap1-NRF2 pathway

**Fig. 3:**
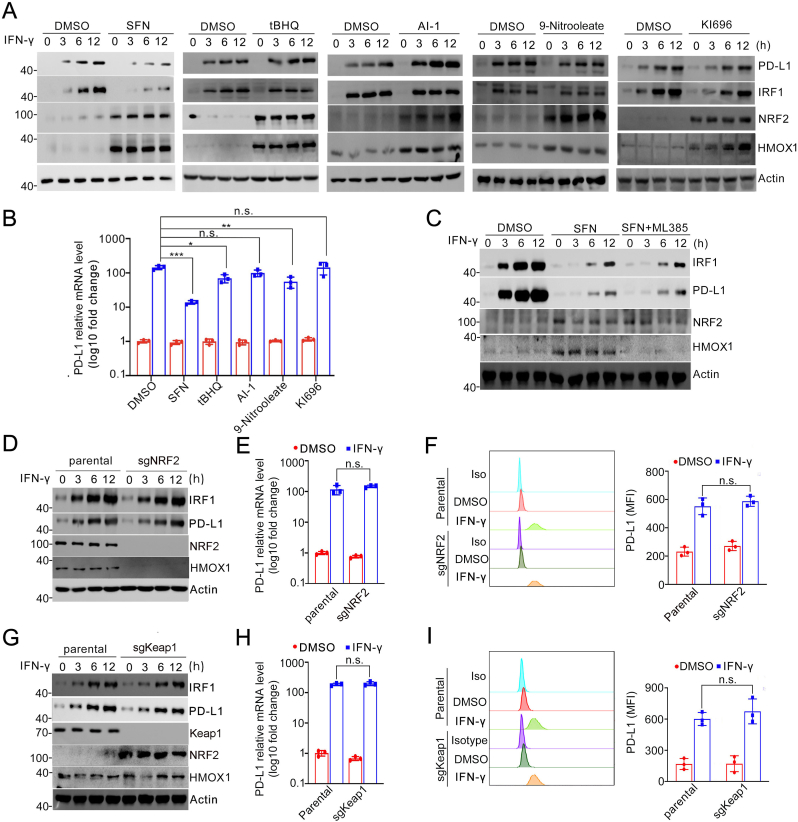
SFN blocks PD-L1 expression independent of its roles towards the Keap1-NRF2 pathway. **A** WB analyses of the indicated proteins in the WCL from A498 cells pretreated with SFN, tBHQ, AI-1, 9-Nitrooleate, KI696 (25 μM) for 2 h and treated with IFN-γ (200 ng/mL) for the indicated times. **B** RT-qPCR analyses of PD-L1 mRNA levels in A498 cells pretreated with SFN, tBHQ, AI-1, 9-Nitrooleate, KI696 (25 μM) for 2 h and treated with IFN-γ (200 ng/mL) for 10 h n = 3. **C** WB analyses of the indicated proteins in A498 cells pretreated with SFN (25 μM) and ML385 (10 μM) for 2 h and treated with IFN-γ (200 ng/mL) for the indicated times. **D** WB analyses of the indicated proteins in parental and NRF2-KO H1299 cells treated with IFN-γ (200 ng/mL) for the indicated times. **E** RT-qPCR analyses of PD-L1 mRNA levels in parental and NRF2-KO H1299 cells treated with IFN-γ (200 ng/mL) for 10 h n = 3. **F** Flow cytometry analyses of surface PD-L1 in parental and NRF2-KO H1299 cells treated with IFN-γ (200 ng/mL) for 10 h n = 3. **G** WB analyses of the indicated proteins in parental and Keap1-KO H1299 cells treated with IFN-γ (200 ng/mL) for the indicated times. **H** RT-qPCR analyses of PD-L1 mRNA levels in parental and Keap1-KO H1299 cells treated with IFN-γ (200 ng/mL) for 10 h n = 3. **I** Flow cytometry analyses of surface PD-L1 in parental and Keap1-KO H1299 cells treated with IFN-γ (200 ng/mL) for 10 h n = 3. Data are shown as means ± SD. The *p* values were calculated using the One-way ANOVA test in (B, E, F, H, I). *∗p* < 0.05, *∗∗p* < 0.01, *∗∗∗p* < 0.001.

Numerous studies have highlighted Keap1 as the primary target of SFN[18]. A previous investigation showed that PD-L1 is a direct transcriptional target of NRF2[19]. Therefore, we investigated to determine whether the impact of SFN on PD-L1 induction is mediated through NRF2 protein stabilization. In addition to SFN, there are numerous electrophilic agents that can disrupt the interaction between NRF2 and Keap1, leading to NRF2 stabilization. We tested several of these agents, including tBHQ, AI-1, 9-Nitrooleate, and KI696[9]. While these agents robustly stabilized and induction of its transcriptional target HMOX1, none of them were able to suppress IFN-γ-inducible PD-L1 expression to the extent observed with SFN (Fig. 3A and B). We also utilized ML385, a highly specific NRF2 inhibitor, and found that while it effectively blocked SFN-induced HMOX1 expression[20], it was unable to block IFN-γ-inducible PD-L1 and IRF1 expression (Fig. 3C). Furthermore, knockout (KO) of Keap1 or NRF2 in A498 cells showed no effect on IFN-γ-inducible PD-L1 and IRF1 expression (Fig. 3D–I). Collectively, our results indicate that SFN blocks IFN-γ-inducible PD-L1 expression through a mechanism independent of the Keap1-NRF2 regulatory axis.

### 2.4 SFN exhibits inhibitory effects on IFN-γ-induced STAT1 activation

**Fig. 4:**
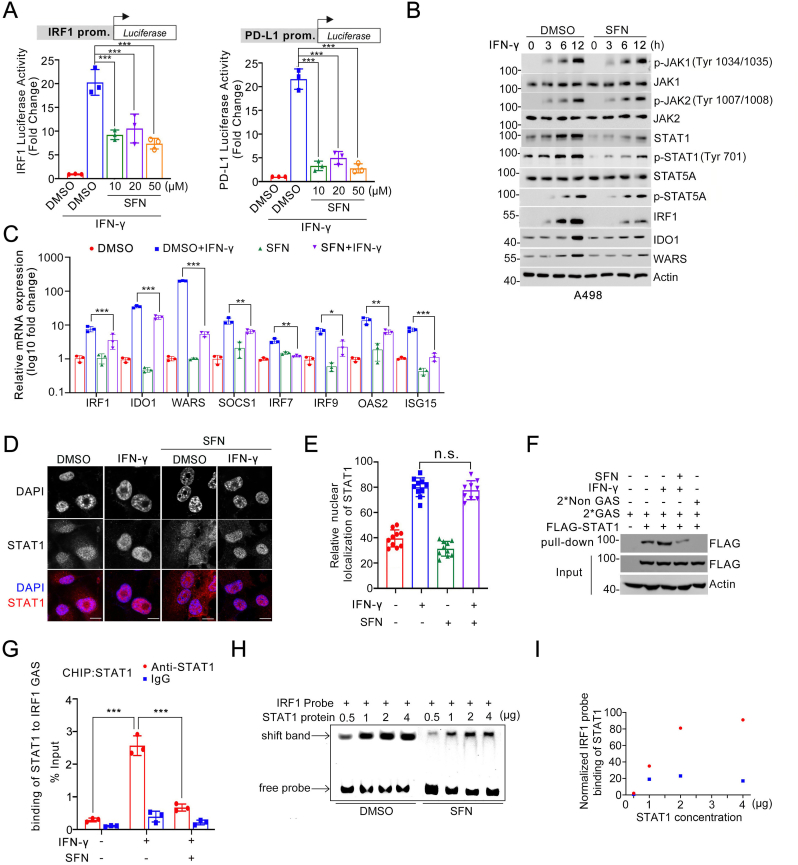
SFN exhibits inhibitory effects on IFN-γ-induced STAT1 activation. **A** Measurement of IRF1 and PD-L1 promoter activity in A498 cells pretreated with SFN (25 μM) for 2 h and treated with IFN-γ (200 ng/mL) for 10 h n = 3. **B** WB analyses of the indicated proteins in A498 cells pretreated with SFN (25 μM) for 2 h and treated with IFN-γ (200 ng/mL) for the indicated times. **C** RT-qPCR analyses of STAT1, IRF1 transcriptional target mRNA levels in A498 cells pretreated with SFN (25 μM) for 2 h and treated with IFN-γ (200 ng/mL) for 10 h n = 3. **D, E** Representative IF images of A498 cells treated with pretreated with DMSO or SFN (25 μM) for 2 h and treated with IFN-γ (200 ng/mL) for 100 min. Cells were stained with STAT1 and DAPI (D). Scale bar, 10 μm. The intensity of STAT1 was quantified using ImageJ (E). n = 10. **F** A498 cells transfected with FLAG-STAT1 were pretreated with DMSO or SFN (25 μM) for 2 h and treated with DMSO or IFN-γ (200 ng/mL) for 100 min. FLAG-STAT1 was immunoprecipitated with biotin-labeled oligonucleotides comprising tandem IRF1 binding sites (2xGAS) or scramble non-GAS sites. 5 % of the input and the immunoprecipitates was subjected to WB analyses with the anti-FLAG antibody. **G** A498 cells were pretreated with SFN (25 μM) for 2 h and treated with IFN-γ (200 ng/mL) for 100 min. Cells were cross-linked, sonicated and immunoprecipitated with IgG or anti-STAT1-specific antibody. The amount of precipitated DNA (IRF1 promoter) was measured by qPCR. n = 3. **H, I** Recombinant protein STAT1 was treated with SFN (25 μM) and then the mixture was incubated with FAM-labeled IRF1 probe and loaded for EMSA analysis. Image was taken using Typhoon (H). Normalized IRF1 probe binding of STAT1 protein were quantified using ImageJ (I). Data are shown as means ± SD. The *p* values were calculated using the One-way ANOVA test in (A, C, E, G). *∗p* < 0.05, *∗∗p* < 0.01, *∗∗∗p* < 0.001.

We observed that SFN effectively inhibited luciferase expression driven by either the IRF1 or PD-L1 promoter in response to IFN-γ treatment (Fig. 4A), indicating that SFN acts upstream to impede IRF1 transcription. Upon IFN-γ treatment, the JAK-STAT signaling pathway plays a central role in the transcriptional upregulation of IRF1. IFN-γ binds to its receptor, resulting in the activation of JAK1/2. Once activated, JAK1/2 phosphorylate STAT1, which subsequently forms a dimer and translocates to the nucleus. In the nucleus, the STAT1 dimer binds to specific DNA sequences known as gamma-activated sequence (GAS) elements within the IRF1 promoter region. This binding then leads to the activation of IRF1 transcription [21]. To determine whether SFN exerts a negative effect on the JAK-STAT pathway, we found that SFN had no impact on the IFN-γ-induced phosphorylation of JAK1, JAK2, and STAT5A in A498 cells. However, SFN markedly compromised the IFN-γ-inducible expression of STAT1 and its phosphorylation by JAK1/2. Additionally, the IFN-γ-inducible expression of other reported STAT1-IRF1 transcriptional targets, such as WARS and IDO1, was also compromised in SFN-treated cells (Fig. 4B). qRT-PCR measurements further demonstrated that SFN markedly reduced the IFN-γ-induced upregulation of multiple downstream targets of STAT1, not limited to IRF1 (Fig. 4C). Nuclear translocation is a critical step in IFN-γ-induced STAT1 activation; however, SFN did not affect the nuclear translocation of STAT1 induced by IFN-γ (Fig. 4D and E). Moreover, we performed DNA affinity binding assays using biotin-labeled oligonucleotides containing GAS motifs to pull down active DNA-binding STAT1. As shown in Fig. 4F, enrichment of STAT1 was significantly decreased in SFN-treated cells. Results from chromatin immunoprecipitation (ChIP)-qPCR assays also demonstrated that SFN markedly decreased the binding affinity of STAT1 to the IRF1 promoter (Fig. 4G). Furthermore, in electrophoretic mobility shift assay (EMSA) experiments, SFN inhibited the binding ability of STAT1 to the IRF1 promoter DNA (Fig. 4H and I). Collectively, our results indicate that SFN inhibits STAT1 activation upon IFN-γ treatment.

### 2.5 SFN covalently modifies cysteine 155 and 174 on the STAT1 protein

**Fig. 5:**
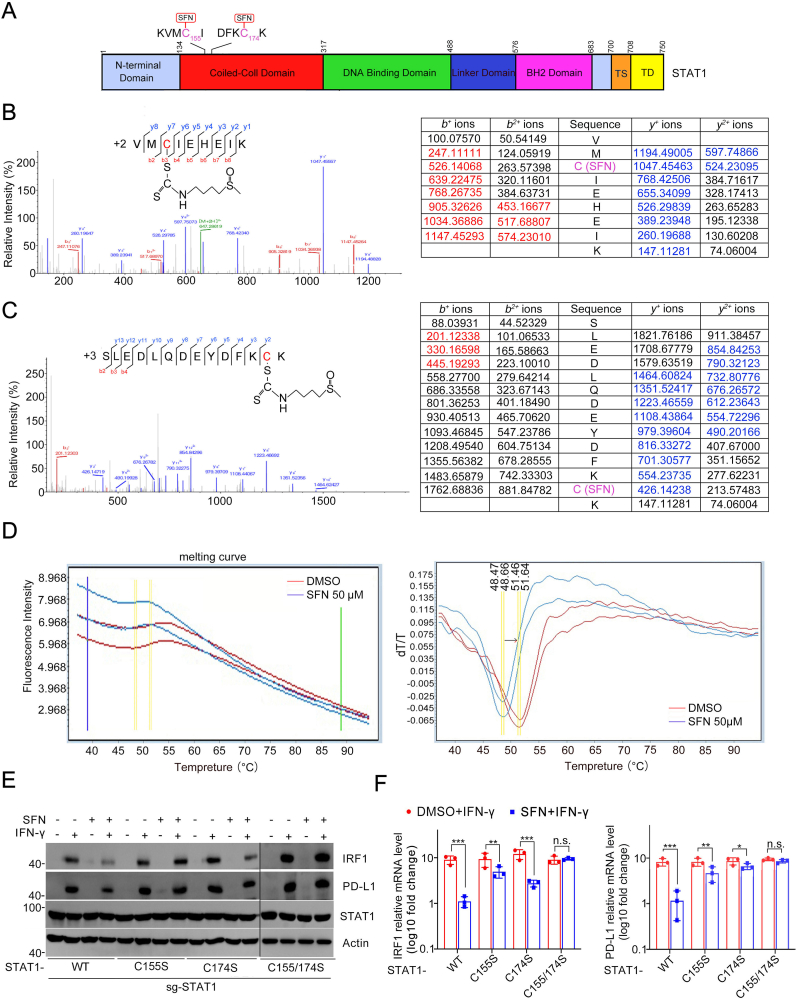
SFN covalently modifies cysteine 155 and 174 on the STAT1 protein. **A** Schematic diagram of STAT1 showing the position of SFN modification at C155 and C174 sites. **B, C** Product ion tandem mass spectra of STAT1 peptide (containing C155 or C174) obtained during data-dependent LC-MS/MS analysis. Data were acquired using high resolution accurate mass measurement, and mass assignments were within 10 ppm of the theoretical values. **D** Recombinant protein STAT1 was treated with SFN (25 μM) and then the mixture was incubated with Sypro Orange dye. The raw fluorescence melts from a 96 well DSF experiment are analyzed (left panel). The first derivative of is calculated to determine T_M_ values (right panel). **E** STAT1 KO A498 cells were generated and then reconstituted with EV, STAT1-WT, or mutant. WB analyses of the indicated proteins in parental A498 cells and stable A498 cells pretreated with SFN (25 μM) for 2 h and treated with IFN-γ (200 ng/ml) for 10 h. **F** STAT1 KO A498 cells were generated and then reconstituted with EV, STAT1-WT, or mutant. RT-qPCR analyses of IRF1 and PD-L1 levels in A498 cells pretreated with SFN (25 μM) for 2 h and treated with IFN-γ (200 ng/mL) for 10 h n = 3. Data are shown as means ± SD. The *p* values were calculated using the One-way ANOVA test in (F). *∗p* < 0.05, *∗∗p* < 0.01, *∗∗∗p* < 0.001.

Previous studies have shown that SFN modifies cysteine 151 on Keap1 and impedes its function [8]. Given this information, we hypothesized that SFN may directly modify cysteine residues on STAT1. To confirm this, we overexpressed FLAG-STAT1 in 293T cells and treated them with SFN. LC-MS/MS analysis of immunoprecipitated FLAG-STAT1 from SFN-treated cells revealed the covalent modification of cysteine 155 and 174 by SFN (+242.15 Da) (Fig. 5A–C). Amino acid sequence alignment indicated that these two cysteine residues of STAT1 are unique compared to other members of the STAT protein family, although they are present in murine STAT1 (Fig. S5A). Protein thermal stability was assessed through differential scanning fluorimetry (DSF) upon SFN binding to STAT1. The thermal unfolding data was analyzed using a two-state unfolding transition model to determine the protein's melting temperature (Tm), which is indicative of protein thermal stability. As shown in Fig. 5D, SFN treatment decreased the Tm of STAT1.

To verify the functional significance of these modified sites, we generated STAT1 KO A498 cells (Figs. S5B and C). STAT1 KO completely abolished IFN-γ-induced PD-L1 and IRF1 expression (Fig. S5D). We then reconstituted STAT1 KO cells with wild-type STAT1 or mutants of STAT1 at cysteine 155 (-C155S), 174 (-C174S), or both (-C155/174S). Notably, we observed comparable induction of IRF1 and PD-L1 upon IFN-γ treatment in these reconstituted cell lines, indicating that these mutations did not impair the transcriptional activity of STAT1 (Fig. S5E). However, the suppression of IRF1 and PD-L1 induction by SFN upon IFN-γ treatment was severely compromised in cells reconstituted with STAT1-C155S, STAT1-C174S, or STAT1-C155/174S mutant (Fig. 5E and F). Collectively, our results indicate that SFN exerts its inhibitory effect on the STAT1-IRF1-PD-L1 regulatory axis through the covalent modification of STAT1 cysteines.

### 2.6 SFN exerts an antitumor immunity effect via suppressing PD-L1 expression

**Fig. 6:**
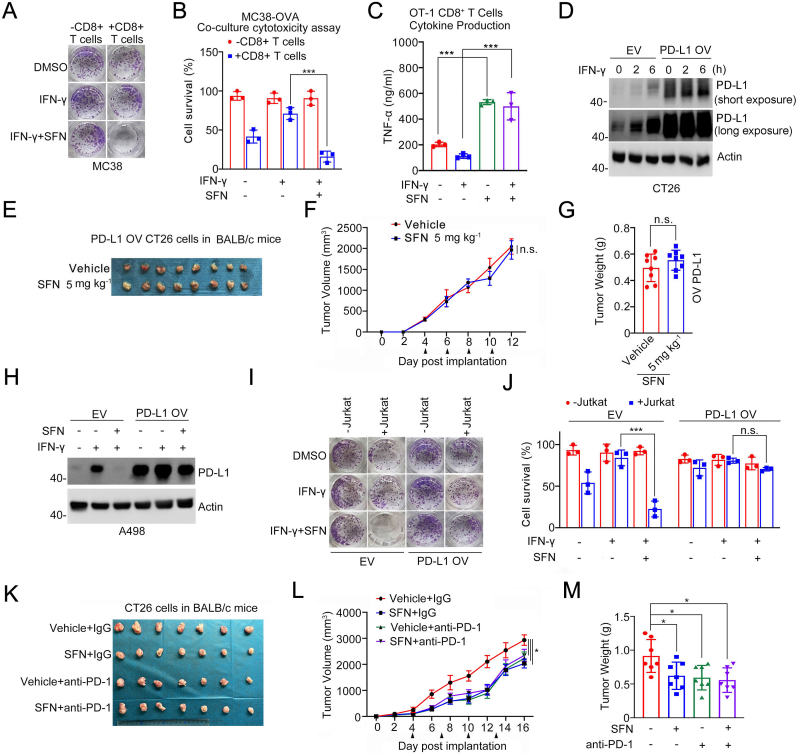
SFN exerts an antitumor immunity effect via suppressing PD-L1 expression. **A, B** CD8^+^ T cell-mediated cancer cell kill assays *in vitro*. OVA-peptide coated MC38 cells were pretreated with SFN (25 μM) for 2 h and then treated with IFN-γ (200 ng/ml) for 10 h. The treated MC38 cells were co-cultured with activated CD8^+^ T cells isolated from OT-1 C57BL/6 mice for 48 h, then subjected to crystal violet staining. MC38 cells to T cells ratio, 1:5. n = 3. **C** Cells co-culture supernatants in (A) were collected and the cytokines were assessed with ELISA kit for testing TNF-α. n = 3. **D** WB analyses of the indicated proteins in CT26 cells stably overexpressing EV or PD-L1 (PD-L1 OV) treated with IFN-γ (200 ng/ml) for 10 h. **E-G** CT26 cells stably overexpressing EV or PD-L1 were injected s.c. into the right flank of BALB/c mice and treated with Vehicle or SFN at different day points. Tumor growth was measured every other day for 12 days. Tumors in each group at day 12 were harvested and photographed (E), tumor volume (F) and tumor weight (G) at each time point was documented. n = 8 (8 mice per group). **H** WB analyses of the indicated proteins in A498 cells stably overexpressing EV or PD-L1 (PD-L1 OV), pretreated with SFN (25 μM) for 2 h and then treated with IFN-γ (200 ng/ml) for 10 h. **I, J** Jurkat T cell-mediated cancer cell killing assay *in vitro*. A498 cells stably overexpressing EV or PD-L1 were pretreated with SFN (25 μM) for 2 h and then treated with IFN-γ (200 ng/ml) for 10 h. The treated A498 cells were co-cultured with Jurkat T cells for 48 h, then subjected to crystal violet staining. A498-to-Jurkat ratio: 1:5. n = 3. **K-M** CT26 cells were injected s.c. into the right flank of BALB/c mice and treated with SFN and anti-PD-1 antibody or IgG isotype control at different day points. Tumor growth was measured every other day for 16 days. Tumors in each group at day 16 were harvested and photographed (K), tumor volume (L) and tumor weight (M) at each time point was documented. n = 7 (mice per group). Data are shown as means ± SD. The *p* values were calculated using the Two-way ANOVA test in (F, L). The *p* values were calculated using the One-way ANOVA test in (B, C, G, J, M). *∗p* < 0.05, *∗∗p* < 0.01, *∗∗∗p* < 0.001.

Given the inhibitory effect of SFN on the STAT1-IRF1-PD-L1 regulatory axis, we investigated whether SFN exerts an antitumor immune effect through impeding PD-L1 expression. To explore this, we utilized chicken ovalbumin (OVA)-specific OT1 C57BL/6 mice, which are transgenic mice expressing a T-cell receptor (TCR) specific to a peptide derived from the OVA protein[22]. This model allows us to study the intricate interactions between the PD-L1 pathway and T cell immunity in a controlled experimental setting. *In vitro*, we prepared OVA-specific OT1 CTLs and murine colon carcinoma MC38 cells coated with OVA peptide. In this assay, we selected MC38 cells because they are derived from C57BL/6 mice, whereas CT26 cells originate from BALB/c mice. Treatment with SFN (25 μM) did not significantly affect the proliferation of OT1 CD8^+^ CTLs after 4 days of treatment, nor did it impact cytokine production by the CTLs (Figs. S6A–C). Furthermore, SFN did not noticeably affect the viability of MC38 cells (Figs. S6D and E). Subsequently, MC38 cells were pretreated with SFN followed by IFN-γ treatment before co-culturing with OT1 CD8^+^ CTLs. Remarkably, SFN treatment significantly enhanced the killing efficacy of OT1 CD8^+^ CTLs against MC38 cells (Fig. 6A and B). Moreover, SFN treatment markedly reduced the levels of secreted TNF-α (an indicator of T cell activation) in the co-culture media (Fig. 6C).

To directly investigate the contribution of PD-L1 in the antitumor effects of SFN in immunocompetent mice, we conducted tumor assays using CT26 cells with stable overexpression of PD-L1 (Fig. 6D). Strikingly, SFN's antitumor effect on CT26 tumors, which exogenously overexpressed PD-L1, was completely abolished (Fig. 6E–G). Similarly, through Jurkat co-culture cytotoxicity assays, we showed that SFN had no noticeable antitumor immune effect on A498 cells with stable overexpression of PD-L1 (Fig. 6H–J). SFN alone did not significantly affect the proliferation of Jurkat cells or the viability of A498 cells (Figs. S6E and F). Furthermore, SFN did not synergistically enhance the antitumor effect of anti-PD-1 antibody, suggesting that SFN and PD-1 blockade likely operate within the same pathway (Fig. 6K–M). Collectively, our results indicate that the antitumor effect of SFN is primarily mediated through the modulation of PD-L1 expression.

### 2.7 The combination of SFN and CTLA-4 blockade effectively suppresses tumor growth

**Fig. 7:**
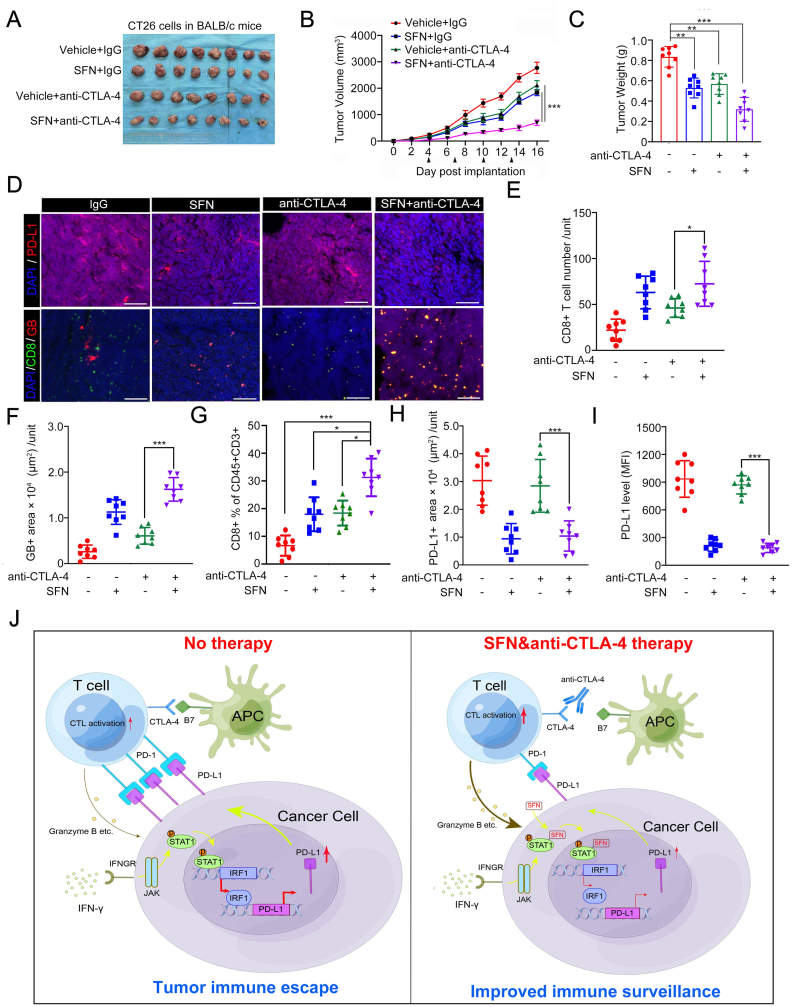
The combination of SFN and anti-CTLA4 effectively suppress tumor growth. A-C CT26 cells were injected s.c. into the right flank of BALB/c mice and treated with SFN and anti-CTLA4 antibody or IgG isotype control at different day points. Tumor growth was measured every other day for 16 days. Tumors in each group at day 16 were harvested and photographed (A), tumor volume (B) and tumor weight (C) at each time point was documented. n = 8 (8 mice per group). **D** Immunostaining of PD-L1, CD8, and Granzyme B in the CT26 tumors (Fig. 7A). DAPI: nuclear. counterstaining. Scale bar, 100 μm. **E-F** Quantification of CD8 and Granzyme B in (Fig. 7A) using imageJ. Unit = 229042 μm^2^. **G** Flow cytometry analysis of CD45^+^CD3^+^CD8^+^ T cell population in the leukocyte fractions from Percoll density gradient separation. n = 8. **H** Quantification of PD-L1 using imageJ. n = 8. Unit = 259042 μm^2^. **I** The cell lysates were extracted from CT26 tumors (Fig. 7A) by Percoll density gradient centrifugation and the surface PD-L1 levels were analyzed by flow cytometry. n = 8. **J** A schematic diagram depicting the proposed mechanism through which SFN promotes antitumor immunity via blocking IFN-γ-induced PD-L1 expression. Data are shown as means ± SD. The *p* values were calculated using the Two-way ANOVA test in (B). The *p* values were calculated using the One-way ANOVA test in (C, E, F, G, H, I). *∗p* < 0.05, *∗∗p* < 0.01, *∗∗∗p* < 0.001.

Recent studies have focused on combining immune inhibitory checkpoints PD-L1 and CTLA-4 blockade to enhance therapeutic efficacy[23]. Building upon our earlier findings, we selected CTLA-4 blockade as the combination therapy for SFN in CT26 tumor model. Strikingly, the combination of SFN and anti-CTLA-4 exhibited significantly better inhibitory effects on CT26 tumors compared to monotherapy, without causing any notable changes in body weight (Fig. 7A–C, Fig. S6G). By the way, we acknowledged that the inhibitory effect of SFN on CT26 tumors was not so impressive compared to the data in Fig. 1A, we believe these differences arise, in part, from inherent batch-to-batch variability. However, this does not affect our overall conclusion. Supporting the mechanistic findings mentioned above, IF analysis of tumor tissues revealed that SFN combined with anti-CTLA-4 led to an increase in CD8^+^ CTL population and GB release levels compared to monotherapy (Fig. 7D–F). By preparing tumor cell suspensions via collagenase digestion of excised tumors, we found that the combination of SFN and anti-CTLA-4 resulted in a more pronounced upregulation of CD8^+^ CTL population within tumor-infiltrating lymphocytes (TILs) compared to monotherapy (Fig. 7G, Fig. S6H). Moreover, we observed that the combination therapy had a similar impact as SFN alone in reducing PD-L1 levels in tumor cells. Treatment with anti-CTLA-4 alone did not significantly affect PD-L1 levels in tumor tissues (Fig. 7H and I). Collectively, our results indicate that SFN has the potential to augment the efficacy of anti-CTLA-4 therapy without additional toxicity.

## 3 Discussion

Derived from cruciferous vegetables, SFN has emerged as one of the most extensively studied ITCs due to its potential health benefits. While more research is needed to fully understand its mechanisms of action and potential therapeutic uses, the evidence so far suggests that SFN could be an important tool in the fight against cancer. suggesting the involvement of the host immune system in SFN-mediated tumor suppression. We illustrate the immunomodulatory role of SFN in reducing PD-L1-mediated immune suppression. We acknowledge that, although we consistently observed the inhibitory effects of SFN on mouse tumors, the extent of inhibition varied across experiments. In Future work, we will continue investigate the antitumor immune effects of SFN across a variety of tumor cell lines in different mouse models.

SFN exhibits multiple molecular mechanisms contributing to its antitumor effect. SFN activates the Keap1-NRF2-ARE pathway, leading to the induction of phase II detoxifying enzymes that neutralize carcinogens and oxidative stress. However, accumulating evidence indicates that NRF2 can act as a double-edged sword, Capable of mediating tumor suppressive or pro-oncogenic functions, depending on the specific biological context of its activation. In principle, the controlled activation of NRF2 might reduce the risk of cancer initiation in normal cells by scavenging ROS and maintaining genomic stability. In contrast, cells that have already undergone transformation and show constitutive or prolonged activation of NRF2 signaling may pose a significant clinical challenge, as they are associated with an aggressive phenotype characterized by therapy resistance and poor prognosis [24]. This is supported by the common incidence of NRF2 activation through Keap1 or NRF2 mutations in human cancers[24], which further fuels the controversy surrounding whether the anti-cancer effect of SFN can be attributed to its regulation of the NRF2 pathway. Additionally, SFN modulates epigenetic alterations to reactivate tumor suppressor genes and silencing oncogene[25]. Other studies have shown that SFN induces cell cycle arrest and apoptosis, inhibits angiogenesis by suppressing VEGF and MMPs, and disrupts aberrantly activated signaling pathways like PI3K/Akt, NF-κB, and Wnt/β-catenin[5]. The immunomodulatory effects of SFN have been extensively reported in various human disease models [4]. For example, SFN stimulates NK cells cytotoxicity and increased the infiltration of lymphocyte T-cells in prostate tumors resulting in a reduction of metastasis, although the detailed molecular mechanisms are still elusive[26]. In this study, we propose that the antitumor effects of SFN are, at least in part, mediated via the host immune system. The expression of PD-L1, one of the major immune checkpoint receptors in tumor cells, can be effectively blocked by SFN through modulating the STAT1-IRF1 axis.

Although the applications of anti-PD1/PD-L1 antibodies in clinical revolutionized cancer treatment, small-molecule inhibiting of PD-L1 expression as a potential therapeutic approach sill offer certain benefits compared to anti-PD-L1 antibody, which is sometimes trigger immune-related adverse events (irAEs) due to nonspecific immunological activation[13]. The anti-cancer potential of SFN has been explored in clinical trials for multiple cancer types. Testing combinations of SFN as an immunotherapy agent, together with other anticancer therapies such as chemotherapy, radiation therapy, and targeted therapies, is warranted in future studies.

In addition to SFN, several small molecules have shown potential antitumor immunity in a PD-L1-dependent manner. Metformin, commonly used to treat diabetes, activates AMP-activated protein kinase, leading to the PD-L1 phosphorylation and subsequent degradation[15]. JQ1, a bromodomain and extraterminal domain (BET) inhibitor, has also been found to disrupt the binding of BET proteins to the PD-L1 gene promoter, preventing its transcriptional activation[27,28]. Silvestrol, a natural compound derived from plants, has demonstrated promising anti-PD-L1 activity. It inhibits the eukaryotic initiation factor 4A (eIF4A)-dependent STAT1 translation, resulting in the suppression of PD-L1 transcription[29]. Collectively, the combined use of these small molecules highlights their potential as therapeutic agents targeting PD-L1 expression, thereby enhancing immune responses against cancer cells. Further research is needed to fully understand their mechanisms and potential clinical applications.

In addition to the well-known target Keap1, the direct substrates targeted by SFN are still poorly known. In this study, we proved evidence that SFN covalently modified STAT1 at two cysteine residues to inhibit STAT1 activation. It is consistent with previous studies showing that cysteine modifications of STAT family proteins play crucial roles in functional regulation. For example, oxidative stress-induced S-glutathionylation of specific cysteine residues in STAT1 and STAT3 led to their inactivation, impairing their transcriptional activity [30]. Conversely, S-nitrosylation of cysteine residues enhances the nuclear translocation and activation of STAT3, promoting its transcriptional activity [31]. Palmitoylation of STAT3 on cysteine 108 promotes its membrane recruitment and phosphorylation[32]. Succination of Cysteine 492 in STAT1 by fumarate, a TCA cycle metabolite had been reported although the functional consequence is still known [33]. Overall, cysteine modifications dynamically regulate the activity, localization, and function of STAT family proteins, playing pivotal roles in various cellular processes and potentially providing therapeutic targets for diseases involving dysregulated STAT signaling pathways. However, we need to emphasize that the target of SFN's immunomodulatory effects should not be limited to STAT1. In our study, we detected cysteine residues (C155 and C174) on STAT1 are modified by SFN *in vitro* though LC-MS/MS methods. Although we demonstrated these two sites are critical for SFN-mediated PD-L1 expression, we did not exclude whether more cysteines *in vivo* that might be affected. In addition, STAT3 can enhance PD-L1 expression in certain inflammatory or oncogenic contexts. it remains to be determined whether STAT3 plays a role in SFN-mediated PD-L1 inhibition and whether it can be directly modified by SFN. These questions warrant further investigation. Hence, the development of chemical tools capable of systematically profiling SFN-mediated cysteine modification of STAT1, and other potential targets in cells with site-specific resolution would greatly aid in thoroughly delineating the antitumor immune functions of SFN.

## 4 Materials and methods

### 4.1 Cell culture, transfection, and viral infection

293T, H1299, DU145, A498, CT26, and LLC and were obtained from the American Type Culture Collection (ATCC). KPIC cells were described in a previous study[34]. H1299, CT26, LLC, and KPIC cells were maintained in DMEM with 10 % (v/v) FBS. DU145 and A498 cells were maintained in RPMI medium with 10 % (v/v) FBS. The authenticity of the cell lines was checked using DNA fingerprinting. The cells were cultured in a humidified incubator at 37 °C with 5 % CO2. We conducted transient transfection using EZ Trans (Shanghai Life-iLab Biotech). For lentivirus transfection, pCDH-based vectors and virus packing constructs were transfected into 293T cells. 48 h after transfection, the virus supernatant was collected. CT26 cells were infected with viral supernatant in the presence of polybrene (8 μg/mL) and then selected in growth media containing 1.5 μg/mL puromycin. To prevent mycoplasma contamination, Plasmocin (InvivoGen) was added to the cell culture media. Regular testing for mycoplasma contamination was performed using the Lookout Mycoplasma PCR Detection Kit (Sigma). The antibodies, reagents, primers, and other resources are listed in Tables S1–3.

### 4.2 Gene KO cell line generation

pX459 plasmid was used to clone guide oligos targeting STAT1, NRF2, or Keap1 gene. A498 cells were plated and transfected with pX459 constructs overnight. 24 h after transfection, cells were screened with puromycin (1 μg/mL) for a duration of 3 days. The surviving cells were then seeded in a 96-well plate using limited dilution to isolate monoclonal cell lines. The KO cell clones were screened using WB analysis, and their validity was confirmed through Sanger sequencing. The specific sequences of the gene-targeting single guide RNAs (sgRNAs) are listed in Table S1.

### 4.3 Western blot

Cell lysates or immunoprecipitates were subjected to SDS-PAGE and the proteins were transferred to nitrocellulose membranes (GE Healthcare). The membranes were blocked in Tris-buffered saline (pH 7.4) containing 5 % non-fat milk and 0.1 % Tween-20, washed twice in TBS containing 0.1 % Tween-20, incubated with the primary antibody for 2 h, and then with the secondary antibody for 1 h at room temperature. The proteins of interest were visualized using ECL chemiluminescence system (Santa Cruz Biotechnology).

### 4.4 RT-qPCR assay

Total RNA was isolated from cells using the TRIzol reagent (Thermo). Subsequently, cDNA was synthesized through reverse transcription using the PrimeScript RT Master kit (TAKARA) following the manufacturer's instructions. PCR amplification was performed using the AceQ Universal SYBR qPCR Master Mix Kit (Vazyme). All quantifications were normalized to the level of endogenous control GAPDH. The primer sequences for the qPCR used are listed in Table S1.

### 4.5 Transcriptional reporter assay

Luciferase assays (Promega) was performed on cells that were transiently transfected with the promoter reporter construct (IRF1/PD-L1-Luc) and pTK-galactosidase plasmids. Luciferase activity in the cell lysates was measured using the luciferase assay system with a Berthold Lumat LB 9507 luminometer (Promega). To establish internal control, luciferase activity was normalized to the activity of galactosidase. Each assay was conducted in triplicate, and the results were verified through at least three independent repeat experiments.

### 4.6 Immunofluorescence assay

For staining cultured cells, the cells were plated on chamber slides and fixed with 4 % paraformaldehyde at room temperature (RT) for 30 min. After rinsing with PBS, the cells were permeabilized with 0.1 % Triton-X100 in PBS for 15 min at RT. Subsequently, the cells were washed with PBST (PBS with 0.1 % Tween-20) and blocked with 5 % donkey serum in PBS for 1 h. Primary antibodies were then added to the cells in PBS and incubated overnight at 4 °C in the dark. Following another round of washing with PBST, fluorescence-labeled secondary antibodies were applied, and DAPI was used for nuclear counterstaining for 1 h at RT in the dark. Finally, the slides were mounted in ProlongGold (Invitrogen). The stained cells were visualized and imaged using a confocal microscope (LSM880, Zeiss) equipped with a 63 × /1.4NA Oil PSF objective.

For staining graft tumor tissues, the tumor tissue was isolated from mice after perfusion with 0.1 M PBS (pH 7.4) and fixed with 4 % paraformaldehyde for 3 days at 4 °C. The fixed tumor tissue was then dehydrated in a 30 % sucrose solution for 2 days. The tumor tissues were embedded in an OCT block and frozen for cryostat sectioning. Cryostat sections with a thickness of 45 μm were washed with PBS and incubated in a blocking solution (PBS containing 10 % goat serum, 0.3 % Triton X-100, pH 7.4) for 2 h at RT. In the antibody reaction buffer (PBS with 10 % goat serum, 0.3 % Triton X-100, pH 7.4), the sections were stained with primary antibodies against CCA3, CD8, Granzyme B, and PD-L1 overnight at 4 °C. Subsequently, Alexa 488, 594, and 647 secondary antibodies were applied at RT for 3 h. Nuclear staining was performed using DAPI. The sections were sealed with an antifluorescence quencher (Abclonal). The stained samples were visualized and imaged using an inverted confocal microscope (Olympus, FV300) with a 40 × objective, capturing images along the z-axis. The intensity of CCA3, PD-L1 and Granzyme B staining was quantified using ImageJ by calculating the total area of positive staining in at least 8 units. The analysis results were obtained in triplicate from three different fields. The numbers of CD8 per unit were calculated.

### 4.7 Mouse tumor implantation assay

The animals were housed in a pathogen-free environment with unrestricted access to food and water. All experimental procedures were approved by the Ethics Review Committee for Animal Experimentation of Fudan University in advance (approval No: TJBG11422102). Female BALB/c mice were obtained from SLAC Laboratory Animal Co., Ltd. CT26 cells (1 × 10^5^) were subcutaneously (s.c.) injected into 6-week-old female BALB/c mice. Eight mice were used for each data point, with one tumor per mouse. SFN dissolved in PEG with PBS or a vehicle (10 % β-cyclodextrin), was administered via intraperitoneal injection three times per week at different doses, starting from 2 days after tumor cell injection. Concurrently, IgG and anti-CTLA4 antibody (200 μg per mouse in 200 μL HBSS saline buffer) were also intraperitoneally administered every three times per week, starting from 2 days after tumor cell injection. Tumor volumes were calculated using the ellipsoid volume formula: V = (L × W^2^)/2, where L represents the length and W represents the width of the tumor. Mice were euthanized when signs of tumor ulceration became evident or when tumors reached the maximum permitted size. Tumor weight was monitored as indicated. Additionally, tumor tissues were divided, with a portion fixed in formalin and embedded in paraffin for IF analysis, with a portion for WB analysis, and another portion used for analysis of TILs by flow cytometry.

### 4.8 Surface PD-L1 expression detection with flow cytometry

Cells were detached from the culture plates using Versene (Thermo), and then resuspended in PBS containing Fc Block (BD Biosciences). The cell suspension was centrifuged, and the cells were incubated for 30 min with a human anti-PD-L1 antibody (Biolegend) and an isotype control. Following the incubation, the cells were washed with PBS to remove unbound antibodies. The stained cells were analyzed using an Fortessa flow cytometer (BD Biosciences). FlowJo software was employed for data analysis. During data collection, debris characterized by low forward scatter (FSC) and side scatter (SSC) was excluded from analysis. Furthermore, single cells that were negative for the live/dead discriminant were gated for further analysis.

### 4.9 LC-MS/MS analysis

Recombinant human STAT1 (Abclonal) was incubated with SFN in 100 μL of 100 mM Tris-HCl buffer (pH7.8) for 2h at RT. Mass spectrometry grade trypsin was added to sample at a trypsin/STAT1 ratio of 1:50 (w/w) and incubated at 37 °C overnight. LC-MS analysis was performed using a nanoflow EASYnLC 1200 system (Thermo) coupled to an Orbitrap Exploris480 mass spectrometer (Thermo). A one-column system was adopted for all analyses. Samples were analyzed on a home-made C18 analytical column (75 μm i.d. × 25 cm, ReproSil-Pur 120C18-AQ, 1.9 μm (Dr. Maisch GmbH). The mobile phases consisted of Solution A (0.1 % formic acid) and Solution B (0.1 % formic acid in 80 % ACN). The derivatized peptides were eluted using the following gradients: 5–8% B in 2 min, 8–44 % B in 38 min,44–70 % B in 8 min, 70–100 % B in 2 min, and 100 % B for 10 min, at a flow rate of 200 nL min. High-field asymmetric-waveform ion mobility spectrometry (FAIMS) was enabled during data acquisition with compensation voltages set as −40 and −60 V.MS1 data were collected in the Orbitrap (60,000 resolution). Charge states between 2 and 7 were required for MS2 analysis, and a 45 s dynamic exclusion window was used. Cycle time was set at 1 s. MS2 scans were performed in the Orbitrap with HCD fragmentation (isolation window 1.6; 15,000 resolution; NCE 30 %, max injection time 30 ms). The results of were processed with UniProt human protein database (75,004 entries) and the using Protein Discoverer(version 2.4, thermo Fisher Scientific)with Mascot (version 2.7.0, Matrix Science). The mass tolerances were 10 ppm for precursor and fragment Mass Tolerance 0.05 Da. Up to two missed cleavages were allowed. Oxidation and cysteine-bound sulforaphane were selected as variable modifications. The proteomics data have been deposited to the ProteomeXchange Consortium via the PRIDE partner repository with the dataset identifier PXD042240.

### 4.10 Pull-down assays of GAS-binding protein complexes

The binding capacity of proteins from A498 cells to a GAS-containing DNA oligonucleotide was assessed using *in vitro* pull-down assays. A498 cells were transfected with FLAG-STAT1 and stimulated with 200 ng/mL IFN-γ or DMSO for 30 min. The cells were then lysed in a 0.1 % NP40 buffer. Following centrifugation, the supernatant was incubated with 1 μM of a biotin-conjugated double-stranded DNA probe containing the GAS sequence of IRF1. Subsequently, the DNA-protein complexes were incubated with 45 μL of streptavidin magnetic beads for an additional 2 h at 4 °C. Following incubation, the DNA-protein complexes bound to the magnetic beads were washed twice using a 0.1 % NP40 buffer supplemented with a cocktail of inhibitors. Finally, the DNA-protein complexes were resolved by SDS-PAGE for further analysis.

### 4.11 ChIP-qPCR assay

The cells were cross-linked by incubating them with 1 % formaldehyde in cell culture media for 20 min at 37 °C. After cross-linking, the cells were washed twice with ice-cold phosphate-buffered saline (PBS) and lysed in SDS buffer. The SDS buffer contained 1 % SDS, 10 mM EDTA, 50 mM Tris-HCl (pH 8.1), and a protease inhibitor cocktail (Selleck). To shear the chromatin, the lysed cells were sonicated on ice using twenty 10-s pulses to obtain DNA fragments ranging from 200 to 800 base pairs. The resulting chromatin supernatants were diluted in a 10-fold dilution buffer composed of 0.01 % SDS, 1.1 % Triton X-100, 1.2 mM EDTA, 16.7 mM Tris-HCl (pH 8.1), and 16.7 mM NaCl. To quantify the amount of input DNA, 20 μL of diluted DNA from each sample were removed. The remaining supernatants were subjected to immunoprecipitation by incubating with antibodies specific to STAT1 or IgG as a control overnight at 4 °C. The protein-DNA complexes were then precipitated using protein A/G-Magnetic beads for 2 h. Samples were then washed consecutively in low salt wash buffer (0.1 % SDS, 1 % Triton X-100, 2 mM EDTA, 20 mM Tris-HCl (pH 8.1), 150 mM NaCl), high salt wash buffer (0.1 % SDS, 1 % Triton X-100, 2 mM EDTA, 20 mM Tris-HCl (pH 8.1), 500 mM NaCl), LiCl wash buffer (0.25 M LiCl, 1 % IGEPAL-CA630, 1 % sodium deoxycholate, 1 mM EDTA, 10 mM Tris-HCl (pH 8.1)), and twice in TE buffer (10 mM Tris-HCl, 1 mM EDTA, pH 8.0). The protein-DNA complexes were then eluted for qPCR analysis.

### 4.12 DSF assay

A total of 2 μg of recombinant STAT1 protein was dissolved in 100 mM Tris-HCl buffer at pH 7.8. SYPRO Orange dye (Thermo) was added to the buffer at an appropriate concentration. A series of reaction mixtures was prepared, each containing the STAT1 protein sample and SYPRO Orange dye (Thermo), with increasing temperatures ranging from 25 to 95 °C. The reaction mixtures were subjected to thermal cycling using a real-time PCR instrument. The temperature was gradually increased, and the fluorescence intensity was continuously monitored as a function of temperature. Measure the fluorescence intensity at each temperature increment. The dye fluorescence increases as the protein unfolds and exposes hydrophobic regions to which the dye binds. The obtained fluorescence intensity data at each temperature point were plotted as a function of temperature to generate a thermal denaturation curve. The curve represents the protein's stability profile, showing changes in fluorescence intensity as the protein undergoes thermal denaturation. The inflection points of the curve, known as the melting temperature (T_m_), represents the temperature at which 50 % of the protein is unfolded. Compare the T_m_ values of different samples to assess protein stability or ligand binding effects.

### 4.13 ELISA assay

Co-cultured cell supernatants were collected, and the cytokines were assessed according to the manufacturer's instructions (BioLegend). Capture antibody solution was added to microplate wells and incubated overnight at 4 °C. Assay diluent buffer was used to block non-specific binding. Serially diluted protein standard or test sample were incubated for 2 h at RT with shaking. The trapped antibody was then added and detected by substrate solution. Optical density of each well was determined within 20 min using a microplate reader set to 450 nm.

### 4.14 T cell-mediated tumor cell killing assay

Briefly, two ovalbumin-transgenic (OT-1) C57BL/6 mice were euthanized. The spleens, as well as the peripheral lymph nodes (cervical, auxiliary, brachial, and inguinal) and mesenteric lymph nodes, were collected and combined. To obtain a single cell suspension, the harvested tissues were gently ground between two rough ends of microscopic slides in PBS. This mechanical disruption facilitated the release of cells from the tissues. The resulting cell suspension contained a mixture of cells from the spleen and various lymph nodes. The resulting CTLs were collected using MojoSort Mouse CD8 T Cell Isolation Kit (Biolegend) and were cultured in RPMI medium with Dynabeads Mouse T-Activator CD3/CD28 (Thermo), IL-2 (10 ng/mL, Stemcell) and stimulated with 10 μg/ml OVA peptide for 3 days. The target mouse cancer cell line MC38, which had been previously plated in a 96-well plate, was coated with 10 μg/ml OVA peptide (SIINFEKL), and incubated at 37 °C for 1 h. Excess OVA peptide was subsequently washed away using RPMI medium. The collected CTLs were added to the plate and co-cultured with the target cancer cells at an effector-to-target (E:T) ratio of 5:1 for 24 h. The medium from the co-culture was collected for ELISA analysis. Additionally, the viable cancer cells were quantified using a spectrometer at OD (570 nm) after crystal violet staining.

To acquire activated Jurkat T cells, Jurkat cells were cultured in RPMI medium with Dynabeads Human T-Activator CD3/CD28 and IL-2 for one week. A498 cells were allowed to adhere to the plates overnight and then incubated for 24 h with activated Jurkat T cells. Jurkat T cells and cell debris were removed by PBS wash, and living cancer cells were then quantified by a spectrometer at OD (570 nm) followed by crystal violet staining.

### 4.15 T cell proliferation assay

Spleen and various lymph nodes were harvested from OT-1 mice. The resulting CTLs were collected using MojoSort Mouse CD8 T Cell Isolation Kit (Biolegend) and were cultured in RPMI medium with Dynabeads Mouse T-Activator CD3/CD28 and IL-2. Activated T cells were stained with 5 μM of carboxyfluorescein diacetate succinimidyl ester obtained from the Cell Division Tracker Kit (Biolegend) and then stimulated with 10 μg/mL OVA peptide. DMSO or SFN was added on day 3 to assess the effects on the proliferation of activated T cell. On day 4, flow cytometry was used to analyze the T cell proliferation compared to the control. Meanwhile, Media samples were collected for ELISA analysis.

### 4.16 CTLs profile analysis by FACS

Excised tumors were digested in collagenase/hyaluronidase (Stemcell) and DNase I (Sigma). Percoll gradient assay (GE Healthcare) was then performed to separate cancer cells and enriched leukocytes. After blocking with CD16/CD32 antibody (Stemcell), cancer cell fractions were stained using PD-L1 antibody (Biolegend). Leukocyte fractions were stained using CD45 (FITC), CD3 (APC), CD8 (PE) antibodies (Biolegend). Stained samples were analyzed using a Fortessa flow cytometer (BD Biosciences).

### 4.17 PD-L1 and PD-1 binding assay

The PD-L1 and PD-1 binding assays was performed essentially as previously described with some modifications [15]. The cells were first fixed with 4 % paraformaldehyde (PFA). Subsequently, the cells were incubated with recombinant human PD-1 Fc protein (R&D Systems). This was followed by incubation with an anti-human Alexa Fluor 488 dye conjugate, which binds specifically to the PD-1 protein. To visualize the cells and their fluorescence, the nuclei were stained with DAPI. The cells were then examined and imaged using a confocal microscope, allowing for the visualization of the fluorescence signal emitted by the Alexa Fluor 488 dye. The fluorescence intensity of Alexa Fluor 488 was subsequently analyzed.

### 4.18 EMSA assay

The STAT1-binding activity of nuclear extracts from SFN treated and non-treated was assayed against the IRF1 promoter sequence. The DNA probe was labeled with FAM. Indicated volumes of recombinant human STAT1 protein were then incubated for 30 min with 10 p.m. of FAM-labeled DNA probe. The protein-DNA complexes were resolved using a native 5 % polyacrylamide gel in 0.5 x TBE. The chemiluminescence was detected by Typhoon system (Amersham).

### 4.19 Statistical analysis

Statistical analysis was performed using GraphPad Prism (GraphPad Software), and the differences between two groups were analyzed using Student's t-test while the differences between multiple groups were analyzed using One-way or Two-way analysis of variance (ANOVA), unless otherwise specified. All data were displayed as means ± S.D. values for experiments conducted with at least three replicates.

## CRediT authorship contribution statement

**Qing Shi:** Investigation, Validation, Visualization, Writing – original draft. **Yajuan Liu:** Investigation, Validation, Visualization. **Wanqi Yang:** Investigation, Validation. **Yao Li:** Supervision. **Chenji Wang:** Conceptualization, Data curation, Funding acquisition, Investigation, Supervision, Writing – original draft, Writing – review & editing. **Kun Gao:** Conceptualization, Funding acquisition, Investigation, Supervision, Writing – original draft, Writing – review & editing.

## Funding

This work was in part supported by the National Natural Science Foundation of China (No. 92357301, 32370726, 91957125, and 81972396 to C.W.; No. 82272992, 91954106, and 81872109 to K.G.; No. 82201571, 32471353 to Q.S.), the State Key Development Programs of China (No. 2022YFA1104200 to C.W.), the Natural Science Foundation of Shanghai (No. 22ZR1449200 to K.G; 22ZR1406600 to C.W.), and the Open Research Fund of State Key Laboratory of Genetic Engineering, Fudan University (No. SKLGE-2111 to K.G.), the Seed Program for Research and Translation of New Medical Technologies, Shanghai Municipal Commission of Health (2024ZZ2016 to K.G.)，the Open Research Fund of Shanghai Key Laboratory of Maternal and Fetal Medicine, Tongji University (No. mfmkf202204 to C.W.), and the Science and Technology Research Program of Shanghai (No. 9DZ2282100).

## Declaration of competing interest

The authors declare that this research was conducted in the absence of any commercial or financial relationships that could be construed as a potential conflict of interest. All authors read and approved the final manuscript.
