# Supplementary material for: The covalent modification of STAT1 cysteines by sulforaphane promotes antitumor immunity via blocking IFN-γ-induced PD-L1 expression
Source: Redox Biol. 2025 Feb 11;81:103543. doi: 10.1016/j.redox.2025.103543 (PMC11875811; doi:10.1016/j.redox.2025.103543)
Supplement: Multimedia component 1 [file mmc1.pdf]

Supplementary Materials for

The Covalent Modification of STAT1 Cysteines by Sulforaphane Promotes Antitumor Immunity

This PDF file includes:

Figure S1. SFN exerts an antitumor activity through the host immune system (related to Figure 1).
Figure S2. SFN blocks PD-L1-inducible IRF1 and PD-L1 induction in multiple cancer cells (related to Figure 2).
Figure S3. The impact of SFN on apoptosis and cell viability in A498 cells (related to Fig. 2).
Figure S4. The impact of ITCs on IFN-γ-inducible IRF1 and PD-L1 induction (related to Figure 2).
Figure S5. SFN covalently modifies cysteine 155 and 174 on the STAT1 protein (related to Figure 5).
Figure S6. SFN exerts an antitumor immunity effect via suppressing PD-L1 expression (related to Figure 6).
Table S1. shRNA/sgRNA sequence information.
Table S2. Primer sequence information.
Table S3. Antibody, recombinant protein, chemicals, and commercial Kit.

Supplementary Figures and Legends

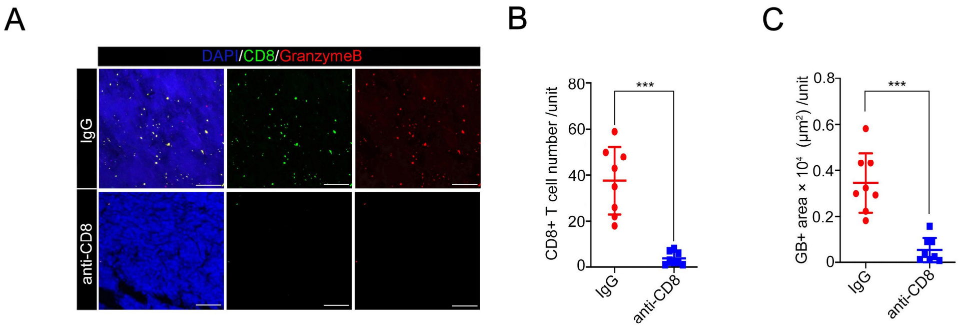

Figure. S1. SFN exerts an antitumor activity through the host immune system (related to Fig. 1).

A Immunostaining of CD8 and Granzyme B in the CT26 tumor mass (Fig. 1g). DAPI: nuclear. Counterstaining. Scale bar, 200 μm. **B** Quantification of CD8 and Granzyme B in (Fig. S1A) using ImageJ. n=8. Unit=631181 μm^2. Data are shown as means ± SD. The *p* values were calculated using the One-way ANOVA test in (B, C). *$p < 0.05$, **$p < 0.01$, ***$p < 0.001$.

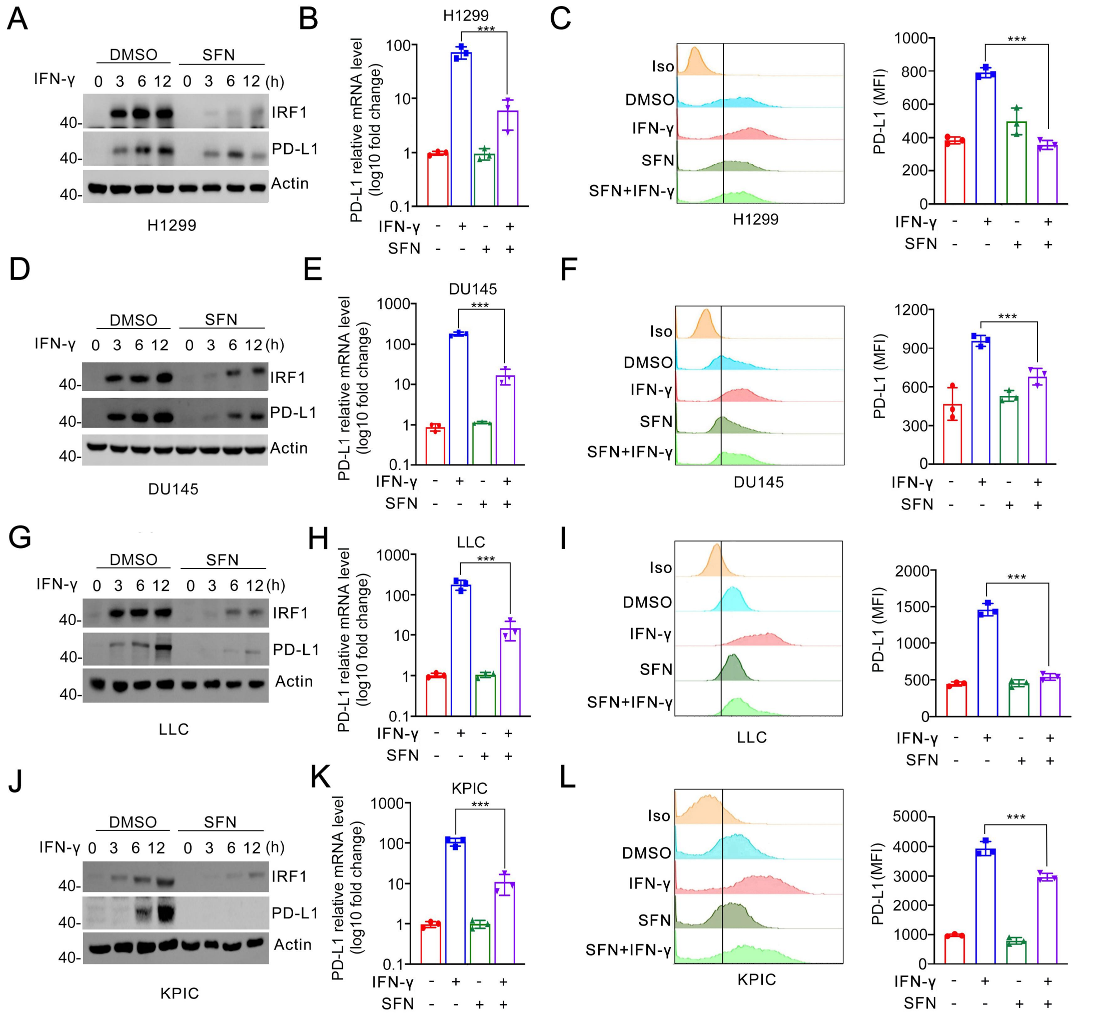

Figure. S2. SFN blocks IRF1 and PD-L1 induction in cancer cells (related to Figure 2).

A WB analyses of the indicated proteins in H1299 cells pretreated with SFN (25 μM) for 2 h and then treated with DMSO or IFN-γ (200 ng/mL) for the indicated times. **B** RT-qPCR analyses of PD-L1 mRNA levels in H1299 cells pretreated with SFN (25 μM) for 2 h and treated with DMSO or IFN-γ (200 ng/mL) for 10 h. n=3. **C** Flow cytometry analyses of surface PD-L1 in H1299 cells pretreated with SFN (25 μM) for 2 h and then treated with DMSO or IFN-γ (200 ng/mL) for 10 h. n=3. **D** WB analyses of the indicated proteins in DU145 cells pretreated with SFN (25 μM) for 2 h and treated with DMSO or IFN-γ (200 ng/mL) for the indicated times. **E** RT-qPCR analyses of PD-L1 mRNA levels in DU145 cells pretreated with SFN (25 μM) for 2 h and treated with DMSO or IFN-γ (200 ng/mL) for 10 h. n=3. **F** Flow cytometry analyses of surface PD-L1 in DU145 cells pretreated with SFN (25 μM) for 2 h and then treated with DMSO or IFN-γ (200 ng/mL) for 10 h. n=3. **G** WB analyses of the indicated proteins in LLC cells pretreated with SFN (25 μM) for 2 h and treated with DMSO or IFN-γ (200 ng/mL) for the indicated times. **H** RT-qPCR analyses of PD-L1 mRNA levels in LLC cells pretreated with SFN (25 μM) for 2 h and treated with DMSO or IFN-γ (200 ng/mL) for

10 h. n=3. **I** Flow cytometry analyses of surface PD-L1 in LLC cells pretreated with SFN (25 μM) for 2 h and then treated with DMSO or IFN-γ (200 ng/mL) for 10 h. n=3. **J** WB analyses of the indicated proteins in KPIC cells pretreated with SFN (25 μM) for 2 h and treated with DMSO or IFN-γ (200 ng/mL) for the indicated times. **K** RT-qPCR analyses of PD-L1 mRNA levels in KPIC cells pretreated with SFN (25 μM) for 2 h and treated with DMSO or IFN-γ (200 ng/mL) for 10 h. n=3. **L** Flow cytometry analyses of surface PD-L1 in KPIC cells pretreated with SFN (25 μM) for 2 h and then treated with DMSO or IFN-γ (200 ng/mL) for 10 h. n=3. Data are shown as means ± SD. The *p* values were calculated using the One-way ANOVA test in (B, C, E, F, H, I, K, L). $^{*}p < 0.05$, $^{**}p < 0.01$, $^{***}p < 0.001$.

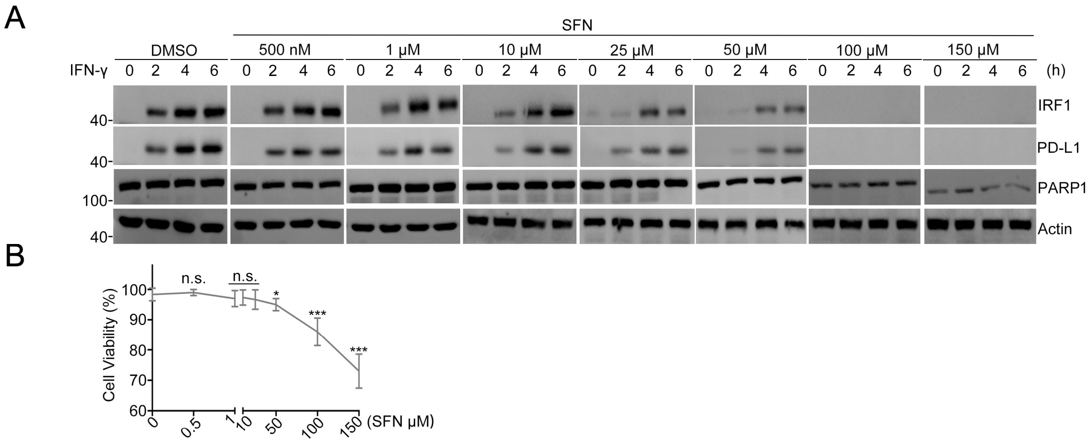

Figure. S3. The impact of SFN on apoptosis and cell viability in A498 cells (related to Fig. 2).

A WB analyses of the indicated proteins in A498 cells pretreated with different doses of SFN for 2 h and treated with DMSO or IFN-γ (200 ng/mL) for the indicated times. **B** CCK-8 assays were conducted to assess the cytotoxic effects of SFN on A498 cells. Data are shown as means ± SD. The p values were calculated using the Two-way ANOVA test in (B). $*p < 0.05$, $**p < 0.01$, $***p < 0.001$.

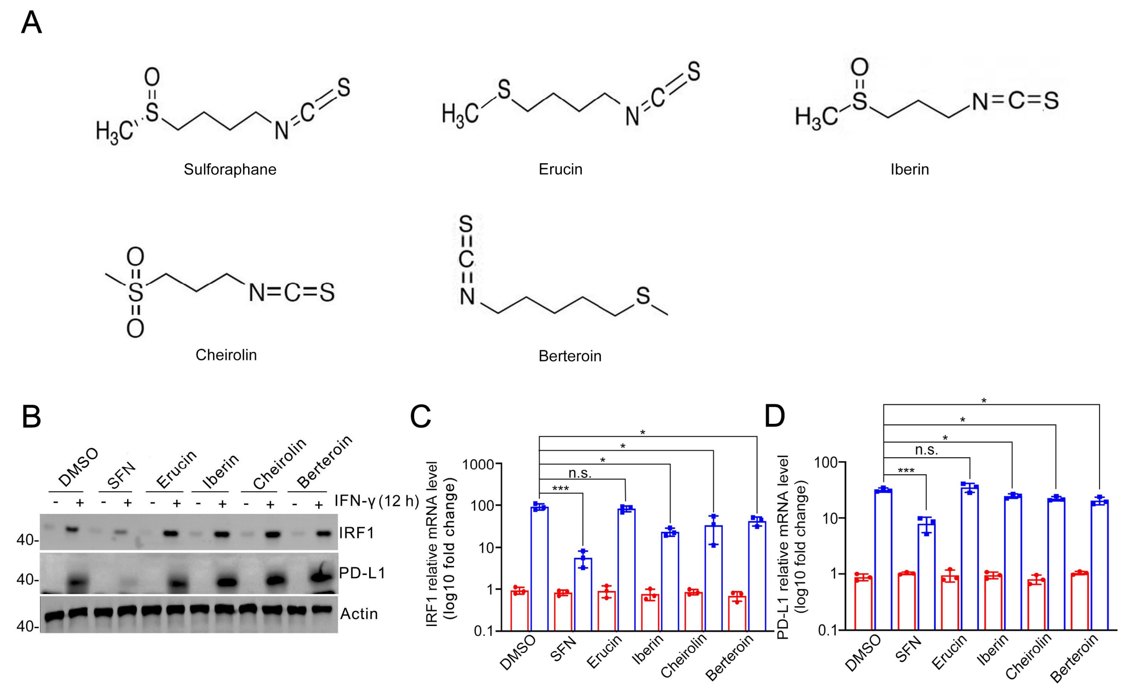

Figure. S4. The impact of ITCs on IFN-γ-inducible IRF1 and PD-L1 induction (related to Fig. 2).

A Chemical structure of naturally occurring ITCs analogous to SFN. **B** WB analyses of the indicated proteins in A498 cells pretreated with different ITCs for 2 h and then treated with DMSO or IFN-γ (200 ng/mL) for 12 h. **C, D** RT-qPCR analyses of IRF1 (C) and PD-L1 (D) mRNA levels in A498 cells pretreated with different ITCs (25 μM) for 2 h and then treated with DMSO or IFN-γ (200 ng/mL) for 10 h. n=3. Data are shown as means ± SD. The p values were calculated using the One-way ANOVA test in (C, D). *$p < 0.05$, **$p < 0.01$, ***$p < 0.001$.

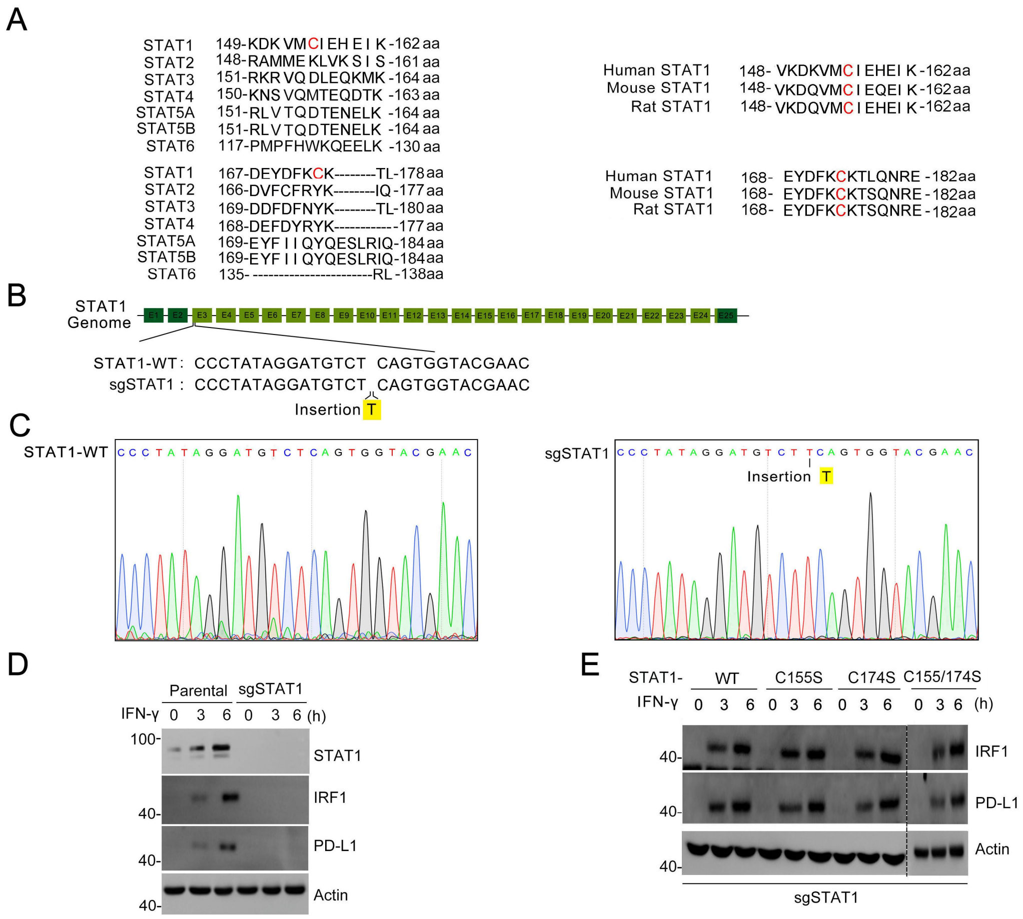

Figure. S5. SFN covalently modifies cysteine 155 and 174 on the STAT1 protein (related to Fig. 5).

A Amino acid sequence alignment of the SFN modified Cysteine sites on STAT1 protein other STAT family proteins. **B** Amino acid sequence alignment of the C155 and C174 on STAT1 homologs in different species. **C** Schematic of CRISPR/Cas9-mediated KO of STAT1 in A498 cells. Sanger sequencing confirming that STAT1 gene was edited in STAT1-KO A498 cells. **D** WB analyses of the indicated proteins in parental or STAT1-KO A498 cells pretreated with SFN (25 μM) for 2 h and then treated IFN-γ (200 ng/mL) for 10 h. **E** WB analyses of the indicated proteins in STAT1 KO A498 cells reconstituted with EV, STAT1-WT, or mutants, treated with IFN-γ (200 ng/mL) for 10 h.

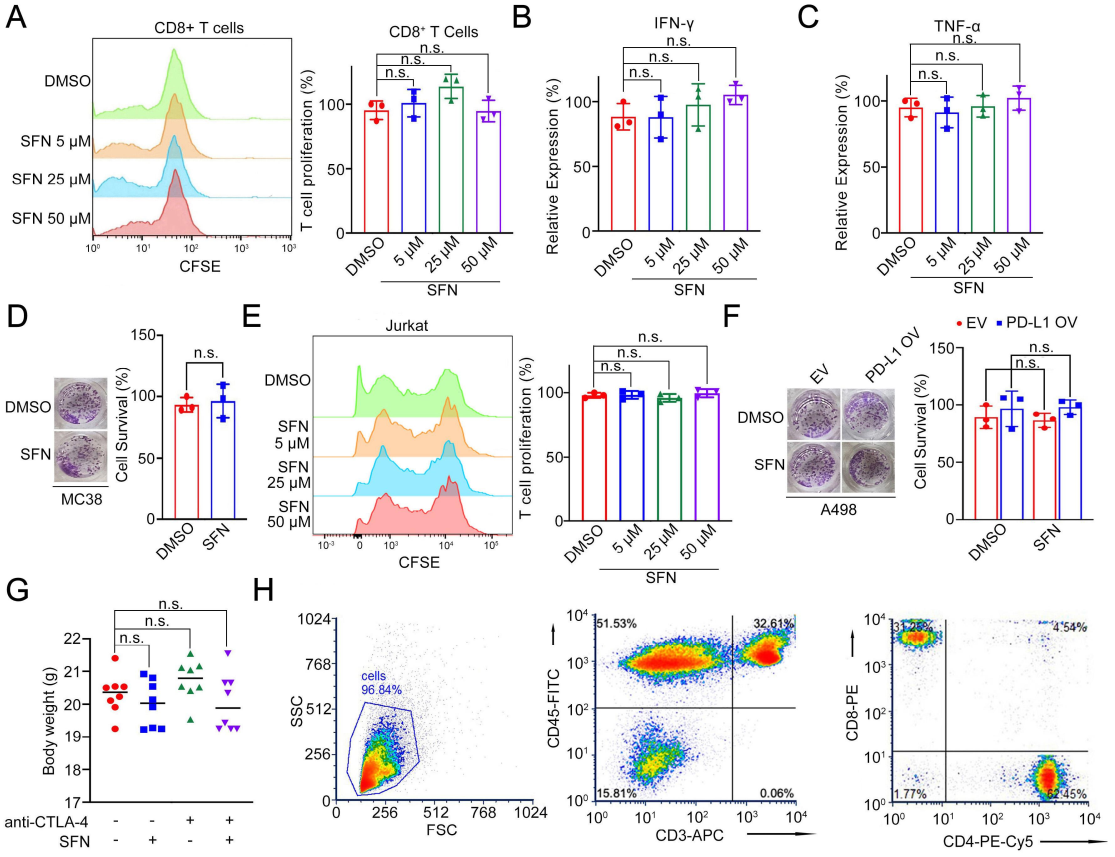

Figure. S6. SFN exerts an antitumor immunity effect via suppressing PD-L1 expression (related to Fig. 6).

A The proliferation of activated T cells from OT-1 mice were treated with different doses of SFN for 24 h and cell proliferation was measured by flow cytometry (left panel). Representative profiles are shown (right panel). n=3. **B-C** Activated T cells from OT-1 mice in (Fig. S6A) were collected. RT-qPCR analyses of TNF-α (B) and IFN-γ (C) mRNA levels in activated T cells. n=3. **D** MC38 cells were treated with DMSO or SFN (25 μM) for 24 h and then subjected to crystal violet staining. n=3. **E** Jurkat cells were treated with DMSO or SFN (25 μM) for 24 h and cell proliferation was measured by flow cytometry (left panel). Representative profiles are shown (right panel). n=3. **F** A498 cells stably overexpressing EV or PD-L1 were treated with DMSO or SFN (25 μM) for 24 h and then subjected to crystal violet staining. n=3. **G** At day 16, tumor mass was excised and its weight was measured after each indicated treatment. n=8. **H** Live cells in leukocyte fraction were gated via FSC and SSC in Fig. 7A. T-cell population was gated based on the surface expression of $CD45^{+}CD3^{+}$. $CD8^{+}$ CTL population was gated based on surface expression of CD8. Quantitation shown in Fig. 7G. Data are shown as means ± SD. The *p* values were calculated using the One-way ANOVA test in (A, B, C, E, F, G). **p* < 0.05, ***p* < 0.01, ****p* < 0.001.

**Supplementary Table.1 Nucleic acid sequence information.**	
Sequences of IRF1 promoter probe 5’-3’ (EMSA)	
GCCTGATTTCCCCGAAATGACGGCAC	
Sequences of 2* GAS probe 5’-3’ (pull down)	
Biotin-TTCCCCGAATTCCCCGAA	
Sequences of 2* non-GAS probe 5’-3’ (pull down)	
Biotin-TTACCCCAATTACCCCAA	
Sequences of sgRNAs	
Gene	Sequence
sgNRF2	TAGTTGTAACTGAGCGAAAA
sgKeap1	AGCCGCCCGCGGTGTAGATC
sgSTAT1	TCCCATTACAGGCTCAGTCG

**Supplementary Table. 2 Primer sequence information.**		
Primers for RT-PCR with cell lines samples		
Gene	F:5'-3'	R:5'-3'
IRF1 (Mouse)	TCCAAGTCCAGCCGAGACACTA	ACTGCTGTGGTCATCAGGTAGG
IRF1 (Human)	GAGGAGGTGAAAGACCAGAGCA	TAGCATCTCGGCTGGACTTCGA
PD-L1 (Mouse)	FGCGGACTACAAGCGAATCACG	CTCAGCTTCTGGATAACCCTCG
PD-L1 (Human)	TGCCGACTACAAGCGAATTACTG	CTGCTTGTCCAGATGACTTCGG
HVEM (Human)	GTGCAGTCCAGGTTATCGTGT	CACTTGCTTAGGCCATTGAGG
CD80 (Human)	AAACTCGCATCTACTGGCAAA	GGTTCTTGTACTCGGGCCATA
FGL-1 (Human)	ATGGCAAAGGTGTTCAGTTTCA	ACAATCTGCATACTGCCTCTTG
CD40 (Human)	ACTGAAACGGAATGCCTTCCT	CCTCACTCGTACAGTGCCA
OX40L (Human)	CCAGGCCAAGATTCGAGAGG	CCGATGTGATACCTGAAGAGCA
4-1BBL (Human)	GGCTGGAGTCTACTATGTCTTCT	ACCTCGGTGAAGGGAGTCC
ICOSL (Human)	GCAGCCTTCGAGCTGATACTC	GTTTTCGACTCACTGGTTTGC
CD70 (Human)	GCAGCCTTCGAGCTGATACTC	GTTTTCGACTCACTGGTTTGC
PD-L2 (Human)	CTCGTTCCACATACCTCAAGTCC	CTGGAACCTTTAGGATGTGAGTG

B7-H3 (Human)	CTGGCTTTCGTGTGCTGGAGA A	GCTGTCAGAGTGTTTCAGAGG C
PVR (Human)	CACTGTCACCAGCCTCTGGAT A	TCATAGCCAGAGATGGATACC TC
VISTA (Human)	AGATGCACCATCCAACTGTGT GG	AGGCAGAGGATTCCTACGAT GC
LGALS 9 (Human)	ACACCCAGATCGACAACTCCT G	CAAACAGGTGCTGACCATCCA C
HLA-A (Human)	ACCCTCGTCCTGCTACTCTC	CTGTCTCCTCGTCCCAATACT
HLA-B (Human)	CAGTTCGTGAGGTTCGACAG	CAGCCGTACATGCTCTGGA
HLA-C (Human)	CCATGAGGTATTTGTGGACCG	TCTCGGACTCTCGTCGTCG
STAT1 (Human)	ACCFCACCTTCAGTCTTTTCC	TGAACTGGACCCCTGTCTTCA
OAS2 (Human)	AAACCAGGCCTGTGATCTTGG	CTATTTCCAGACAACGCCTCC
SOCS1 (Human)	CCGACAATGCAGTCTCCACA	CGAACGGAATGTGCGGAAGT
IRF7 (Human)	AGCTGTGCTGGCGAGAAGG	GCTTGGAGTCCAGCATGTGT
IRF9 (Human)	TCCTCCAGAGCCAGACTACT	CAATCCAGGCTTTGCACCTG
IDO1 (Human)	GCCTGATCTCATAGAGTCTGG C	TGCATCCCAGAACTAGACGTG C
WARS (Human)	GGACATCATCGCCTGTGGCTT T	AGTCGCTGTCAGTGAAGCCGA A
ISG15 (Human)	CTCTGAGCATCCTGGTGAGGA A	AAGGTCAGCCAGAACAGGTC GT
GAPDH (Human)	TGCACCACCAACTGCTTAGC	GGCATGGACTGTGGTCATGAG
GAPDH (Mouse)	CATCACTGCCACCCAGAAGAC TG	ATGCCAGTGAGCTTCCCGTTC AG
Primers for ChIP-qPCR		
gene	F:5'-3'	R:5'-3'
IRF1-GAS	GAGCAGCCGCCCTGTACTT	ACCGAGCAATCCAAACACTT

**Supplementary Table. 3 Antibodies, recombinant proteins, chemicals, and Kits.**					
**No**	**Antibody/ Chemicals**	**Species**	**Cat. No**	**Source**	**Application/ Dilutions**
1	STAT1	Rabbit	14994	CST	IB: 1:1000
2	IRF1	Rabbit	ab186384	Abcam	IB: 1:1000
3	PD-L1	Rabbit	13684	CST	IB: 1:1000
4	Keap1	Rabbit	8047	CST	IB: 1:500
5	NRF2	Rabbit	12721	CST	IB: 1:1000
6	FLAG	Mouse	M185-7	MBL	IB: 1:3000
7	p-STAT1	Rabbit	9167	CST	IB: 1:4000
8	STAT5A	Mouse	4807	CST	IB:1:1000
9	p-STAT5A	Rabbit	4322	CST	IB:1:1000
10	JAK1	Rabbit	3344	CST	IB:1:1000
11	p-JAK1	Rabbit	74129	CST	IB:1:1000
12	JAK2	Rabbit	3230	CST	IB:1:1000
13	p-JAK2	Rabbit	3776	CST	IB:1:1000
14	IDO1	Rabbit	13268-1-AP	Proteintech	IB:1:1000
15	WARS	Rabbit	Ab109213	Abcam	IB:1:1000
16	HMOX1	Rabbit	26416	CST	IB: 1:2000
17	Actin	Rabbit	AC028	Abclonal	IB: 1:10000
18	Granzyme B	Rabbit	17215	CST	IF: 1:200
19	Cleaved CASP3	Rabbit	9664	CST	IF: 1:100
20	CD45 (FITC)	Rat	103108	Biolegend	Flow Cyt: 1:200
21	CD3 (APC)	Rat	100236	Biolegend	Flow Cyt: 1:200
22	CD8 (PE)	Rat	100708	Biolegend	Flow Cyt: 1:200
23	Mouse CD3	Rat	100202	Biolegend	IF:1:50
24	Mouse CD8	Rabbit	Ab217344	Abcam	IF:1:100
25	Mouse PD-L1 (PE)	Rat	124307	Biolegend	Flow Cyt: 1:200
26	Human PD-L1 (PE)	Mouse	329706	Biolegend	Flow Cyt: 1:200
27	PD-1 Fc protein	Human	1086-PD	R&D Systems	
28	Mouse PD-L1	Rabbit	A19135	ABclonal	IF: 1:100
29	Mouse IgG1 Isotype Ctrl	Mouse	981804	Biolegend	Flow Cyt: 1:200
30	Rat IgG1 isotype Ctrl	Rat	400408	Biolegend	Flow Cyt: 1:200
31	Anti-rabbit IgG (Alexa Fluor 488)	Rabbit	4412	CST	IF: 1:2000
32	Anti-Rat IgG (Alexa Fluor 647)	Rat	4418	CST	IF: 1:2000

33	HRP Goat Anti-Mouse IgG	Goat	AS0032	ABclonal	IB: 1:8000
34	HRP Goat Anti-Rabbit IgG	Goat	AS014	ABclonal	IB: 1:8000
35	InVivoMAb anti mouse CTLA4	Mouse	BE0285	BioXcell	
36	InVivoMAb mouse IgG2b isotype control	Mouse	BE0032	BioXcell	
37	InVivoMAb anti mouse PD-1	Mouse	BE0146	BioXcell	
38	IFN-γ	Human	GMP-11725-HNAS	Simo Biological	
39	IFN-γ	Mouse	RP01070	Abclonal	
40	IL-2	Mouse	78081.1	Stemcell Technologies	
41	IL-2	Human	78220.2	Stemcell Technologies	
42	Dynabeads™ Mouse T-Activator CD3/CD28	Mouse	11452D	Thermo	
43	Dynabeads™ Human T-Activator CD3/CD28	Human	11161D	Thermo	
44	CFSE Cell Division Tracker Kit		423801	Biolegend	
45	Collagenase/ Hyaluronidase		07902	Stemcell Technologies	
46	DNase I		10104159001	Sigma-Aldrich	
47	LEGEND MAX™ Mouse IFN-γ ELISA Kit		430807	Biolegend	
48	LEGEND MAX™ Mouse TNF-α ELISA Kit		430907	Biolegend	
49	Pierce ™ Protein A/G Magnetic Beads		20423	Thermo	
50	MojoSort Mouse CD8 T Cell Isolation Kit		480035	Biolegend	

51	ImmunoCult-XF T Cell Expansion Medium	Human	10981	Stemcell Technologies	
52	TNF-α	Human	S7074	Selleckchem	
53	STAT1	Human	RP01251	Abclonal	
54	Recombinant human PD-1 Fc protein	Human	1086-PD-01M	R&D Systems	
55	Percoll		17-0891-09	GE Healthcare	
56	PrimeScript RT Master Mix		RR036Q	TAKARA	
57	AceQ Universal SYBR qPCR Mater Mix Kit		Q511-02/03	Vazyme	
58	Bio-Glo Luciferase Assay System		G7940	Promega	
59	polybrene		TR-10030G	EMD Millipore	
60	puromycin		Anr-pr-2	InvivoGen	
61	EZ Trans		AC04L071	Shanghai Life-iLab Biotech	
62	cocktail		B14001	Selleck	
63	TRIzol		15596018	Thermo	
64	Nitrocellulose membranes		10600002	GE Healthcare	
65	NcmECL High		P2300	NCM Biotech	
66	ProlongGold Antifade Mountant		P36934	Invitrogen	
67	Anti-Mouse CD16/CD32 Antibody		60161.1	Stemcell Technologies	
68	Sulforaphane (SFN)		S5771	Selleckchem	
69	Erucin		4430-36-8	Santa Cruz	
70	Iberin		505-44-2	Santa Cruz	
71	Cheirolin		505-34-0	Santa Cruz	
72	Berteroin		4430-42-6	Santa Cruz	

IB:Immunoblot; IF:Immunoflurence; Flow Cyt:Flow Cytometry; IHC:Immuobiochemistry
